# Multiscale simulations reveal the role of PcrA helicase in protecting against spontaneous point mutations in DNA

**DOI:** 10.1038/s41598-023-48119-z

**Published:** 2023-12-08

**Authors:** Max Winokan, Louie Slocombe, Jim Al-Khalili, Marco Sacchi

**Affiliations:** 1 Leverhulme Quantum Biology Doctoral Training Centre, University of Surrey, Guildford, GU2 7XH UK; 2 School of Chemistry and Chemical Engineering, University of Surrey, Guildford, GU2 7XH UK; 3 School of Mathematics and Physics, University of Surrey, Guildford, GU2 7XH UK

**Keywords:** DNA, Biophysical chemistry, DNA repair enzymes

## Abstract

Proton transfer across hydrogen bonds in DNA can produce non-canonical nucleobase dimers and is a possible source of single-point mutations when these forms mismatch under replication. Previous computational studies have revealed this process to be energetically feasible for the guanine-cytosine (GC) base pair, but the tautomeric product (G$^*$C$^*$) is short-lived. In this work we reveal, for the first time, the direct effect of the replisome enzymes on proton transfer, rectifying the shortcomings of existing models. Multi-scale quantum mechanical/molecular dynamics (QM/MM) simulations reveal the effect of the bacterial PcrA Helicase on the double proton transfer in the GC base pair. It is shown that the local protein environment drastically increases the activation and reaction energies for the double proton transfer, modifying the tautomeric equilibrium. We propose a regime in which the proton transfer is dominated by tunnelling, taking place instantaneously and without atomic rearrangement of the local environment. In this paradigm, we can reconcile the metastable nature of the tautomer and show that ensemble averaging methods obscure detail in the reaction profile. Our results highlight the importance of explicit environmental models and suggest that asparagine N624 serves a secondary function of reducing spontaneous mutations in PcrA Helicase.

## Introduction

The reliable nature of the DNA base pairing rules discovered by Franklin, Watson, and Crick^1,2^ is crucial to maintaining accurate replication of genetic material. A low but finite rate of DNA mutations allows for the gradual evolution of the genome, as well as causing genetic diseases. Spontaneous mutations can occur when the canonical Watson and Crick (WC) pairing is disturbed, causing a mismatch to be formed. Such a mistake can be made permanent through replication, thus altering the genome.

During replication, duplex DNA is unwound and has its strands separated to form two single-stranded templates by a helicase enzyme. Each template is assembled into a new double-stranded molecule via a polymerase enzyme. Polymerase enzymes have remarkable reading fidelity and error-correction mechanisms and are crucial in ensuring genome stability. The reliable nature with which guanine binds to cytosine, and adenine to thymine, is at the core of guaranteeing this stability. Mismatches such as guanine-thymine (G-T) do not form a Watson and Crick-like dimer and are thus rejected by the polymerase^3,4^. However, proton transfers can create rare tautomers that may evade these cellular error-detecting facilities by mimicking the WC geometry.

**Figure 1:**
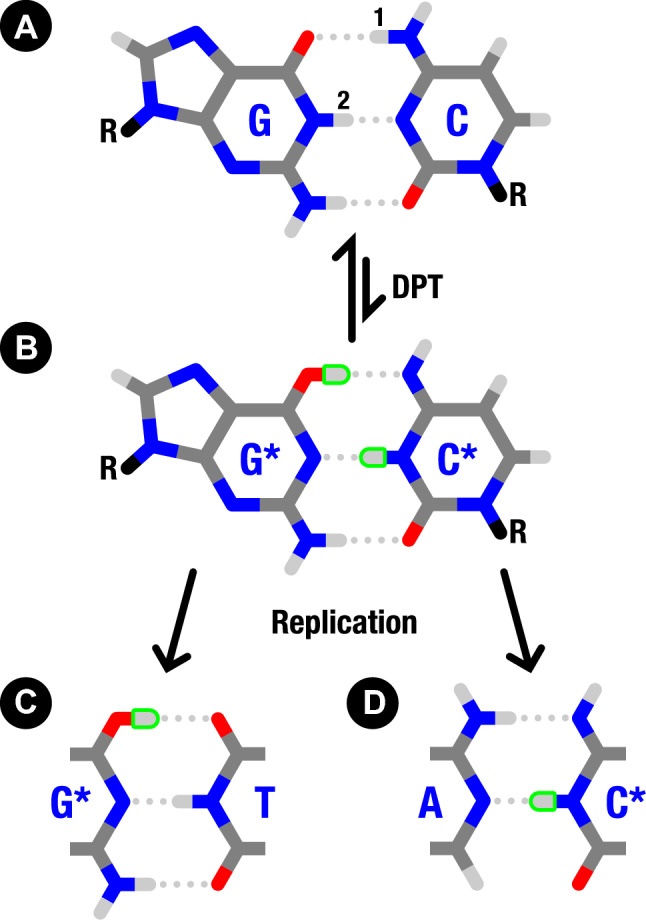
A mechanism for creating spontaneous point mutations in which a canonical GC base pair (**A**) undergoes a double proton transfer (DPT) to an intermediate G$^*$C$^*$ tautomer (**B**) before a helicase enzyme separates the dimer allowing for the creation of the G$^*$-T (**C**) and A-C$^*$ (**D**) mismatches by a polymerase enzyme. Highlighted in green are the transferred hydrogen atoms/protons forming the nonstandard structures. In the canonical GC base pair (**A**) the two protons involved in the DPT are labelled “1” and “2”.

One pathway for creating point mutations is proton transfer (PT) across the nucleotides’ hydrogen bonds. For example, in its canonical (standard) configuration, the guanine-cytosine (GC) dimer is in amino-keto form. A double proton transfer (DPT) across two of its three hydrogen bonds results in a metastable state known as a tautomer and is written as G$^*$C$^*$. The reaction from GC (amino-keto) to G$^*$C$^*$ (imino-enol) is shown in Fig. 1.

The tautomer G$^*$C$^*$ causes both nucleotide bases to have an altered hydrogen bonding profile, which no longer matches their canonical counterparts. This modified steric profile causes problems during DNA replication as a tautomer in the template strand will not match using the WC pairing, instead leading to mismatches. The tautomeric form, G$^*$C$^*$ is ’Watson-Crick’-like because it leaves the secondary structure of the DNA duplex unaffected and can thus evade error-correction mechanisms. While a guanine nucleobase in the template strand would canonically be matched with a cytosine base, the imino form of guanine (G$^*$) pairs instead with thymine, causing a point mutation in the genome as the letter C is replaced with T. Similarly, for the cytosine (enol) tautomer, the genetic letter G is replaced by A.

Pivotal to the feasibility of the WC-like point mutation hypothesis is that the tautomeric dimer can survive in the noisy cellular environment at biological temperatures—and crucially—in the presence of the replisome enzymes. Furthermore, significant nuclear quantum effects have been demonstrated for this reaction, indicating that quantum tunnelling facilitates the DPT in DNA^5,6^ and thus may contribute to genomic variation^7^.

Recent Density Functional Theory (DFT) calculations and hybrid quantum mechanics/molecular mechanics (QM/MM) calculations have revealed the energy landscape governing the DPT in canonical DNA base pairs. Several computational studies of DPT in DNA base pairs show that the contribution of DPT to the production of rare tautomeric forms of DNA base pairs is negligible in AT but not in GC due to the differences in their reaction energy profile. The shallow local minimum corresponding to the tautomeric state of AT present in the potential energy surface^8–13^ vanishes when accounting for free energy corrections^10^. Consequently, AT has been dismissed as a candidate for spontaneous point mutations^14,15^. In GC dimers, the depth of the tautomeric well has been studied with^12^ and without^12,16^ free energy considerations. However, in the free energy surface, the tautomeric well is, crucially, still present^10^. Therefore the GC base pair is the more likely candidate for single-point mutations via the tautomerisation process^5^. Typically, other authors have quoted activation energies in the range 0.42–0.63 eV and reaction asymmetries of 0.29–0.54 eV for the DPT in GC, and both synchronous and asynchronous transfers have been observed^17–20^. The preference for the two protons to transfer in a stepwise fashion appears with more realistic models of the environment that include larger duplex DNA structures such as Angiolari et al.^20^ and Gheorghiu et al.^21^.

Whether the tautomers produced by DPT can have a lasting biological impact by forming WC-like mismatches depends on the role of the replisome in their formation and stability^5,7,17,21,22^. But, to date, no computational investigation has characterised the DPT in the presence of replisome enzymes. This work aims to determine the plausibility of DPT in a helicase-DNA complex.

For replication, the DNA has to unwind and the two strands need to separate. These tasks are performed in cooperation by the topoisomerase and helicase enzymes. One of the most studied and thus best-understood helicase enzymes is that of the gram-positive bacterium named the plasmid copy-reduced (PcrA) helicase. The PcrA helicase belongs to the helicase superfamily 1 and contains a single protein chain, with 649 amino acids forming a monomer with four domains of interest. There have been several experimental and computational studies of the PcrA helicase, revealing stepping motor dynamics^23–28^, roles of individual residues in the ssDNA binding site^29^, and the details of the ATP cleavage cycle^30^.

**Figure 2:**
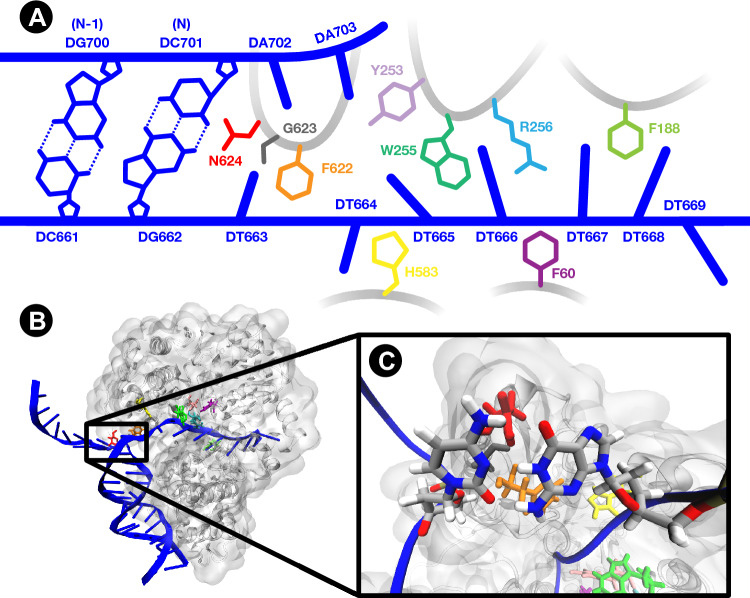
The simulation system for studying proton transfer within the replisome environment comprises the PcrA helicase (grey) in a complex with two DNA strands (blue). (**A**) Shows a schematic of the interface between DNA and the single-stranded DNA binding site of the protein. The final two base pairs of the duplex DNA, pair N (DG662-DC701) and pair N-1 (DC661-DG700), and note-worthy amino acids of the enzyme are highlighted. (**B**) Shows a 3D rendering of the enzyme-DNA complex. (**C**) Is a 3D render zoomed in on the DC701-DG662 QM region within the enzyme-DNA complex where proton transfer is investigated.

The topology of the PcrA resembles a torus (see panel B of Fig. 2), with a single strand of DNA being translocated through, pushing apart the duplex. Figure 2 shows the single-stranded DNA (ssDNA) binding site of PcrA helicase and the end of the DNA duplex. Slocombe *et al.*^18,31^ proposed that DNA bases about to be split by the helicase enzyme encounter a unique environment that modifies the energetics of the tautomerism but did not include an explicit enzyme model. The last base pair of the duplex (base pair N with residues DG662 and DC701) is the last chance for the DPT to occur in the PcrA Helicase complex before the strands are separated. In this work, we model the DPT within a GC base pair embedded in two contrasting biological scenarios: (1) in aqueous duplex DNA and (2) at the entrance to the ssDNA binding site of PcrA helicase, in order to reveal the effect an explicit replisome environment has on the DPT. This work aims to untangle the mechanical and chemical interactions between the helicase and the DNA bases. Additionally, we will investigate the effect of the hydrogen bond stretching on the proton transfer.

## Results

We employ QM/MM to model the proton transfer reaction profile between the hydrogen bonds of DNA. Within the QM/MM framework, we use umbrella sampling (US) to obtain the reaction free energy from an ensemble of steered dynamical trajectories where the system is harmonically restrained to a given reaction coordinate value. Details on the restraints are given in sections 1.1-1.4 and Fig. S1 of the supplementary information (SI). In sections 2.1, 2.2 and Figs. S4, S5 of the SI we validate our level of theory. Initially, we use US on two distinct environments with contrasting environmental complexity. Firstly, we model the aqueous DNA system where the bases of interest are embedded within a stack of bases above and below and surrounded by water and counter ions. Secondly, we model the double proton transfer in GC within an explicit replisome environment (see Fig. 2). For both cases the DNA sequence is given in Table S1 of the SI.

We perform calculations for the duplex’s base pair closest to the helicase (N), and for the rung before (N−1), and at two distinctive timescale limits to home in on the role of the distance between DNA and helicase during DNA strand separation. In the US simulations, it is assumed that all the vibrational degrees of freedom, with the exception of the reaction coordinate, are allowed to relax during the proton transfer. The US trajectories provide a fully equilibrated model to the proton transfer dynamics because every point on the sampled path is able to equilibrate along the degrees of freedom perpendicular to the reaction coordinate. Conversely, we investigate an approximation in which the proton transfer occurs near-instantaneously (i.e. via fast quantum tunnelling), and thus, the surrounding environment is effectively frozen. In the GC dimer the proton transfer is dominated by tunnelling^6^, and atomic rearrangement can play a role in priming a GT mismatch into a ’tunnelling-ready state’^32^. The DPT is likely to occur over a timescale in between the two models, there is a competition between the strand separation and the proton transfer. For both the aqueous dsDNA and DNA-Helicase complex, we have obtained potential energy surfaces for the instantaneous double proton transfer and the minimum energy pathways. Further details are provided in Section 1.5 and Figs. S9–S14 of the SI.

**Figure 3:**
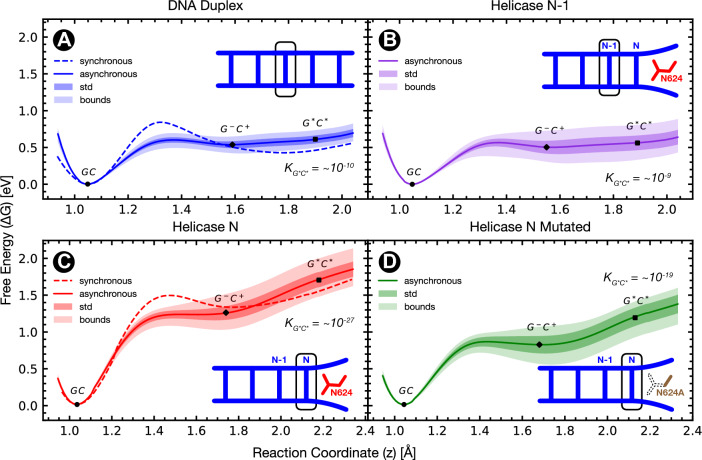
Potentials of mean force (PMF) for the asynchronous double proton transfer in guanine-cytosine obtained from QM/MM Umbrella Sampling (US) simulations for Aqueous Duplex DNA (**A**) and two base pairs in the wild type PcrA Helicase-DNA Complex, pair N−1 (**B**) and pair N (**C**). Panel (**D**) additionally shows the umbrella sampled DPT PMF for the PcrA Helicase with the N624A mutation. For aqueous DNA panel (**A**) and Helicase base pair N panel (**C**) the associated potential of mean force for the concerted DPT is shown as a dashed curve. The concerted barriers with full bootstrapping statistics can be found in Fig. S7 of the supplementary information (SI). Table S2 of the SI gives the sampling times for each of these PMFs. For each panel the equilibrium constant of the tautomeric state $K_{\text {G}^*\text {C}^*}$ is superimposed. In all the US profiles presented here the motion along four distance reaction coordinates (donor-hydrogen and hydrogen-acceptor for each of the two hydrogen bonds) was projected onto a single reaction coordinate using the average relative change from the canonical minimum see Fig. S1 and section 1.4 of the SI.

**Table 1:** Statistical properties of umbrella sampling (US) potentials of mean force (PMF) for the double proton transfer in guanine-cytosine for two pathways: ’synchronous’ where both protons transfer simultaneously, and ’asynchronous’ where the middle proton between the two nitrogen atoms transfers first.

Synchronous US	Property	DNA Duplex	Helicase N-1	Helicase N	Helicase N624A
G$^*$C$^*$ $\downarrow$ GC	$\Delta G _{\text {rxn}}$(eV)	$0.47\pm 0.06$	$0.48\pm 0.22$	$1.35\pm 0.13$	–
	$\Delta G_{\text {fwd}}$(eV)	$0.85\pm 0.20$	$0.56\pm 0.20$	$1.53\pm 0.09$	–
	$\Delta G_{\text {rev}}$(eV)	$0.41\pm 0.21$	$0.08\pm 0.24$	$0.18\pm 0.15$	–
	Reverse barrier (%)	100	100	100	–
	*K*	$2.50\cdot 10^{-8}$	$1.72\cdot 10^{-8}$	$1.46\cdot 10^{-22}$	–

Asynchronous US	Property	DNA Duplex	Helicase N-1	Helicase N	Helicase N624A
GC $\rightarrow$ G*C*	$\Delta G _{\text {rxn}}$(eV)	$0.60\pm 0.05$	$0.55\pm 0.16$	$1.68\pm 0.10$	$1.18\pm 0.13$
	*K*	$1.68\cdot 10^{-10}$	$1.50\cdot 10^{-9}$	$6.70\cdot 10^{-28}$	$9.53\cdot 10^{-20}$
GC $\downarrow$ G^-^C^+^	$\Delta G _{\text {rxn}}$(eV)	$0.55\pm 0.05$	$0.49\pm 0.13$	$1.22\pm 0.09$	$0.82\pm 0.10$
	$\Delta G_{\text {fwd}}$(eV)	$0.60\pm 0.04$	$0.57\pm 0.09$	$1.24\pm 0.07$	$0.86\pm 0.07$
	$\Delta G_{\text {rev}}$(eV)	$0.06\pm 0.03$	$0.09\pm 0.06$	$0.05\pm 0.05$	$0.05\pm 0.04$
	Reverse barrier (%)	100	91	42	68
G^-^C^+^ $\downarrow$ G$^*$C$^*$	$\Delta G _{\text {rxn}}$(eV)	$0.06\pm 0.04$	$0.05\pm 0.06$	$0.46\pm 0.03$	$0.35\pm 0.04$
	$\Delta G_{\text {fwd}}$(eV)	$0.06\pm 0.04$	$0.05\pm 0.06$	$0.46\pm 0.03$	$0.35\pm 0.04$
	$\Delta G_{\text {rev}}$	–	$0.04\pm 0.06$	–	–
	Reverse barrier (%)	0	25	0	0

Reaction energies ($\Delta G _{\text {rxn}}$), forward and reverse energy barriers ($\Delta G_{\text {fwd}}$ and $\Delta G_{\text {rev}}$, respectively), and equilibrium constants (*K*) are reported for each reaction transition and the different replication scenarios studied in this work. DNA duplex refers to the DPT in aqueous linear double-stranded DNA. Helicase N−1 refers to the DG700-DC661 base pair of DNA in complex with PcrA Helicase, and Helicase N to base pair DG662-DC701 of the same complex. N624A refers to the mutation of asparagine N624 into alanine, which does not form hydrogen bonds.

**Figure 4:**
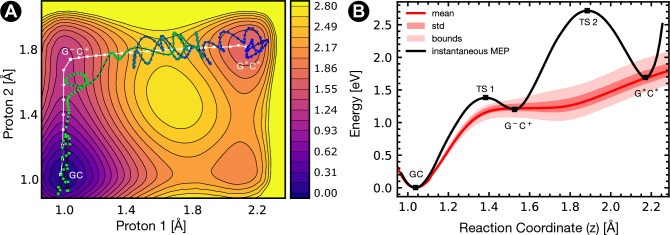
Instantaneous DPT in a snapshot of the PcrA Helicase-DNA complex. (**A**) Contoured heatmap illustrating the DPT’s instantaneous energy surface, the minimum energy pathway within this landscape (white line with circular dots), and the decay path from G$^*$C$^*$ to GC (black line with multicoloured circles). For the decay path, the dots are coloured according to the simulation time, with blue corresponding to the start of the simulation and green at the end of the decay. The colour scale provides the potential energy in eV. (**B**) The free energy of the DPT in an ensemble from US (red curve and red shaded regions) compared to the minimum energy path for DPT within the instantaneous energy landscape (black curve). The instantaneous MEP in panel (**B**) corresponds to the energy profile taken along the MEP in panel (**A**).

**Table 2:** Statistical properties of minimum energy path profiles for the instantaneous double proton transfer in guanine-cytosine for two asynchronous DPT pathways: via the two zwitterions G${}^{-}$C${}^{+}$ and G${}^{+}$C^-^.

Asynchronous MEP	Property	DNA Duplex	Helicase N	Helicase N624A
GC $\rightarrow$ G*C*	$\Delta E_{\text {rxn}}$ (eV)	$1.13\pm 0.25$	$1.84\pm 0.27$	$1.69\pm 0.23$
GC $\downarrow$ G^-^C^+^	$\Delta E_{\text {rxn}}$ (eV)	$0.61\pm 0.17$	$1.35\pm 0.15$	$1.19\pm 0.11$
	$\Delta E_{\text {fwd}}$ (eV)	$0.77\pm 0.23$	$1.38\pm 0.15$	$1.23\pm 0.11$
	$\Delta E_{\text {rev}}$ (eV)	$0.15\pm 0.11$	$0.05\pm 0.05$	$0.04\pm 0.01$
	Reverse barrier (%)	100	80	100
	Product Lifetime (fs)	$200\pm 270$	$30\pm 10$	-
G^-^C^+^ $\downarrow$ G$^*$C$^*$	$\Delta E_{\text {rxn}}$ (eV)	$0.52\pm 0.13$	$0.43\pm 0.17$	$0.50\pm 0.12$
	$\Delta E_{\text {fwd}}$ (eV)	$0.70\pm 0.17$	$1.41\pm 0.18$	$1.23\pm 0.35$
	$\Delta E_{\text {rev}}$	$0.18\pm 0.10$	$0.98\pm 0.09$	$0.73\pm 0.23$
	Reverse barrier (%)	100	100	-
	Product Lifetime (fs)	$120\pm 150$	$260\pm 150$	–
GC $\downarrow$ G${}^{+}$C${}^{-}$	$\Delta E_{\text {rxn}}$ (eV)	–	$1.93\pm 0.34$	$2.04\dagger$
	$\Delta E_{\text {fwd}}$ (eV)	–	$2.24\pm 0.17$	$2.41\dagger$
	$\Delta E_{\text {rev}}$ (eV)	–	$0.32\pm 0.17$	$0.38\dagger$
	Reverse barrier (%)	–	100	100
	Product Lifetime (fs)	–	–	–
G${}^{+}$C${}^{-}$ $\downarrow$ G$^*$C$^*$	$\Delta E_{\text {rxn}}$ (eV)	–	$0.04\pm 0.03$	$-0.15\dagger$
	$\Delta E_{\text {fwd}}$ (eV)	–	$0.60\pm 0.02$	$0.39\dagger$
	$\Delta E_{\text {rev}}$	–	$0.56\pm 0.00$	$0.54\dagger$
	Reverse barrier (%)	–	100	100
	Product Lifetime (fs)	–	–	–

Reaction energies ($\Delta E_{\text {rxn}}$), forward and reverse energy barriers ($\Delta E_{\text {fwd}}$ and $\Delta E_{\text {rev}}$, respectively) are reported for each reaction transition and duplex DNA and the PcrA Helicase-DNA complex. The minimum energy path included the G${}^{+}$C${}^{-}$ Zwitterion in two out of seven replicas with the Helicase and zero out of seven replicas for DNA. Properties of the energetic minima for each replica instantaneous surface are provided in Table S3 of the supplementary information. $\dagger$ are derived from a single replica.

**Figure 5:**
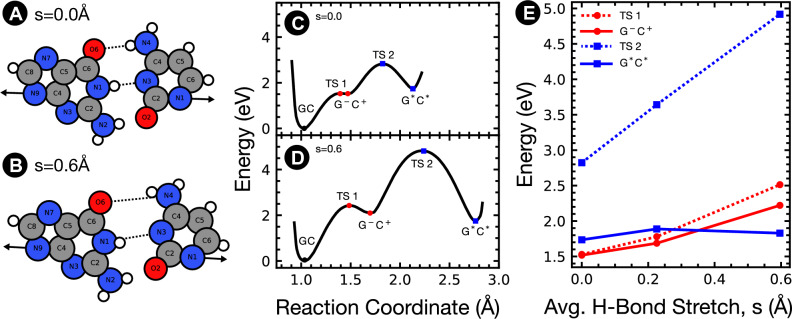
Scheme for determining the effect of mechanical separation on the instantaneous double proton transfer (DPT) energy landscape. Panel (**A**) shows the equilibrium distance GC dimer taken as the starting point for the steered molecular dynamics (SMD). This GC dimer forms the QM region within the base pair N PcrA helicase-DNA complex system as used elsewhere in this work. Two black arrows denote the atoms the constant non-equilibrium pulling force was applied to during the SMD and the direction. The DPT was sampled across the two hydrogen bonds denoted by dotted lines. Panel (**B**) contains the same elements as Panel (**A**), but for a later snapshot in the SMD corresponding to a separation of 0.60 Å. Panel (**C**) shows the minimum energy path (MEP) through the instantaneous potential energy landscape (PES) for the equilibrium separation snapshot. Panel (**D**) is the equivalent of Panel (**C**) for 0.60 Å separation. Panel (**E**) shows the energies of the zwitterion (G${}^{-}$C${}^{+}$), tautomer (G$^*$C$^*$), and transition states at separation values relative to the canonical GC of 0.23 Å and 0.60 Å.

We start with our control system, which is a simulated double-stranded DNA in an aqueous solution within QM/MM. We use this as a baseline for understanding the effect of the helicase on proton transfer and as it allows us to compare to previous studies^19,21,33^.

We determine two categories of free energy reaction paths: asynchronous and synchronous. A synchronous path is one in which both protons transfer simultaneously, whereas an asynchronous path corresponds to the situation in which the middle proton between the two nitrogen atoms transfers first (see Fig. S2 of the SI). The properties of the free energy reaction paths obtained from US are shown in Table 1. These two mechanisms align with those proposed by Gheorghiu et al.^19,21^ who suggested that the reaction proceeds via a statistical mixture of the two paths. The asynchronous pathway is also in agreement with the Path Integral Molecular Dynamics (PIMD) US results of Angiolari, et al.^20^. Other studies suggest that during the dissociation of the bases, the asynchronicity of the proton transfer increases^18,31^.

Here, the reaction asymmetry ($\Delta G _{\text {rxn}}$) for the GC $\rightarrow$ G$^*$C$^*$ process in aqueous DNA is $0.54\pm 0.08$ eV, in good agreement with other aqueous GC dimer studies^10,15,18,34^. Results from Angiolari, *et al*’s PIMD US^20^ include a reaction asymmetry of $0.61\pm 0.02$ eV for an isolated GC dimer, and $0.83\pm 0.01$ eV for an explicitly solvated three base pair DNA duplex for the DPT. The higher asymmetry in the larger hydrated system is attributed by the authors to the presence of a water molecule near the O6 acceptor of the guanine, which in our work is observed in the PcrA-helicase complex (see Discussion). Furthermore, the lower activation energy ($\Delta G_{\text {fwd}}$) for the asynchronous mechanism ($0.60\pm 0.04$ eV) compared to the synchronous mechanism ($0.85\pm 0.20$ eV) suggests that the former is kinetically preferred. For the cases of aqueous duplex DNA and base pair N in complex with PcrA Helicase the potentials of mean force for the synchronous transfer are shown in Fig. 3. Synchronous proton transfer PMFs are shown with statistical uncertainty in Fig. S7 of the supplementary information, including the base pair N−1 case. Further determination of the most favourable DPT pathways are explored using minimum energy paths later in this article. For the asynchronous path, we report a small but clear free energy barrier between the canonical (GC) and zwitterionic (G${}^{-}$C${}^{+}$) states and a barrier-less transition between G${}^{-}$C${}^{+}$ and G$^*$C$^*$ see Table 1 and Fig. 3. Here, the error bars arise from the statistical ensemble of replicas.

We will now turn our attention to the case when the DNA is in contact with the larger replisome environment. Initially, we focus on the end of the double-stranded duplex, that is in close contact with the enzyme’s binding site, since this is the last base pair to potentially undergo proton transfer before entering the helicase. The two base pairs of interest are the duplex’s second nearest base pair (N-1) and the final base pair in the stack (denoted N). These two base pairs are shown in panel A of Fig. 2. Under the initial inspection of Fig. 3, it appears that when DNA forms a complex with PcrA helicase, the free energy surfaces for both the synchronous and asynchronous DPT are significantly different from that of the DNA duplex. Conversely, Fig. 3 and Table 1 show that the single GC base pair between N−1 and the helicase ssDNA binding site sufficiently mimics the duplex DNA environment, resulting in the aqueous DNA and helicase N−1 cases being nearly indistinguishable. Here the aqueous DNA and helicase N−1 cases have a forward reaction barrier and reaction free energy within each other’s uncertainty for both the synchronous and asynchronous proton transfer mechanisms. This result is consistent with other works^35^ demonstrating that three base pairs of DNA are sufficient to model stacking effects on DPT in double-stranded DNA when considering proton transfer in the middle base pair. Consequently, in the N−1 case, the base pair above appears to “shield” the interaction of the helicase from the proton transfer mechanism by providing a local environment closely resembling duplex DNA. Whether by mechanical constraints, induced immobility and conformational deformation induced by electrostatic interactions with the helicase, the G$^*$C$^*$ tautomer in base pair N−1 is protected from the destabilising effect observed in base pair N.

Base pair N, situated at the ssDNA binding site of PcrA helicase, is affected by $\pi$-$\pi$ interactions from only one side (from the base pair below) and is near the side chain of an asparagine amino acid (N624) of the helicase enzyme, shown in red in Fig. 2. The GC base pair N has also been partially opened as the hydrogen bonds have been stretched by 0.3 Å relative to the equilibrium in both the DNA and helicase N−1 cases. Thus, for base pair N, the energetics of the proton transfer is radically changed. The overall endothermicity of the reaction has increased, causing the G$^*$C$^*$ and G${}^{-}$C${}^{+}$ states to be less likely to be populated compared to the aqueous DNA case. The resulting proton transfer profiles indicate that the G*C* state is thermodynamically unstable since it sits on a steep free energy slope which suggests the system is most likely to return to the canonical reactant. In addition, the G${}^{-}$C${}^{+}$ intermediate becomes metastable with only 42% of bootstrapping profiles exhibiting a small reverse barrier back to the canonical GC. Previous work has attributed the greatest effect of a helicase enzyme on the DPT in GC to be due to separation of the base pair which reduces the rate of proton transfer. In our US of the DPT in base pair N of the PcrA complex, the purely mechanical effect of the helicase is incorporated in the QM/MM model by taking snapshots from MD. For base pair N we find the base pair to be separated by an additional 0.15 Å with respect to the aqueous DNA trajectories. According to purely mechanical models^18,31^ a 22% increase int the forward reaction barrier and 1% decrease in the reaction asymmetry are expected at this distance. Our US indicates corresponding changes of 280% to the forward barrier and 180% for the reaction energy. We thus conclude that mechanical separation alone is not enough to describe the changes to the proton transfer energy profile in the presence of the helicase, as neither the radical increase in the forward barrier nor asymmetry between duplex DNA and base pair N in the helicase-DNA complex can be accounted for by existing separation models.

We further investigate the interaction of helicase and the proton transfer mechanism by homing in on the closest residue of helicase. The role of the asparagine in destabilising G${}^{-}$C${}^{+}$ and G*C* is revealed by the US results for the N624A mutated case. Here, asparagine (N624) has been substituted with an alanine (N624A), which has a much smaller (hydrophobic) side chain and cannot form hydrogen bonds with the DNA. As asparagine N624 is not part of the ssDNA binding site of PcrA helicase, which plays a key role in the inchworm mechanism of PcrA^29,36^, its substitution with alanine should only negligibly affect the enzyme’s function. This is further corroborated by the presence of various amino acid motifs in sites 622–626 (see Section 1.8 and Table S4 of the SI), indicating that these residues are not key to PcrA’s primary biological function in strand separation through ssDNA translocation. The precise impact of the N624A mutation on the efficacy of the helicase would need to be experimentally determined. When the alanine mutation is present, some of the destabilising effects of the helicase on G${}^{-}$C${}^{+}$ and G*C* are mitigated. The reaction asymmetry of the G$^*$C$^*$ product relative to GC is reduced by 30%, and a slight reverse barrier between GC $\rightarrow$ G${}^{-}$C${}^{+}$ appears 62% more frequently. In addition to any electrostatic differences due to removing the asparagine’s sidechain, the canonical GC state was also found to be more structurally compressed with the N624A mutation relative to the wild type. During the QM/MM steered MD sampling of the canonical GC within the N624A mutated helicase, the hydrogen bonds were stretched by 0.16 Å relative to equilibrium duplex DNA.

In summary, within our model, we can reproduce the energetic landscape for the aqueous duplex DNA and match it with prior literature^19,21^. We significantly expanded the system’s complexity and fidelity with respect to the biological environment by including the helicase enzyme and determined that the helicase radically alters the free energy landscape suppressing the probability of proton transfer in GC. Furthermore, we found that modifying a single local residue reduces the electrostatic interaction between the helicase and the DNA bases about to dissociate, thus leading to a significant reduction in reaction energy. Overall, this indicates that the helicase environment suppresses the formation of the tautomer and increases the probability of the canonical form of DNA as the strands separate.

We now consider a scenario in which there is not a sufficient amount of time for the system to relax due to very fast DNA strand separation; instead, the system’s vibrational degrees of freedom perpendicular to the reaction coordinate remain “frozen” during the proton transfer. We propose a method to explore the minimum energy pathway that instantaneously transferring protons would take between canonical and tautomeric GC. This allows for the determination of the quantum tunnelling pathway as suggested by previous studies of the DPT in the GC base pair and aqueous DNA^5,6,20,21^, and provides an explanation for the picosecond stability of the tautomer despite the lack of a reverse energy barrier in the US PMF. Here we assume that the helicase is poised to dissociate the base pair N DNA rung and consider the instantaneous proton transfer energy landscape. A similar non-equilibrium model has been applied to the aqueous GC and the GT wobble mismatch in the thumb domain of DNA Polymerase where we showed that nuclear quantum effects play a critical role in the proton transfer reaction of isolated GC bases^6,32^, leading to a fast proton exchange. Accordingly, we expect both the quantum and classical rate of proton transfer to be significantly modified by the increased reaction energies in the DNA-Helicase complex. In this scenario, it is pertinent to consider a GC $\rightarrow$ G$^*$C$^*$ reaction whose product state is solely populated via a fast proton transfer mechanism. In this case, the system will not be subject to a thermalised ensemble energy barrier, but to an instantaneously “frozen” energy landscape with all other degrees of freedom constrained. We obtained multiple frozen potential energy surfaces (PES) for a sample of (canonical GC) QM/MM US snapshots and determined QM/MM single point energies (SPEs) for a two-dimensional grid of proton positions (see Section 1.5 of the SI). Within these landscapes, the locations of local minima were determined using the BFGS optimiser, and the minimum energy path (MEP) between the canonical GC and tautomeric G$^*$C$^*$ was determined using a nudged elastic band (NEB) algorithm. These methods are described in secton 1E of the supplementary information. The MEP is shown as the white line on panel A of Fig. 4, and the energy profile along this MEP is overlaid on the corresponding US result in panel B of Fig. 4. Table 2 displays the properties of the reaction profile along the MEP through the PES for aqueous duplex DNA, base pair N in the Helicase-DNA complex with the asparagine N624 wild type, and alanine N624A mutation. The instantaneous PES model produces comparable reaction asymmetries for each of the studied systems, but find higher transition state energies resulting in increased stability of the rare zwitterionic and tautomeric states. We propose that these raised transition states are due to conformational changes which accommodate the non-canonical protonation states which are populated in US but are frozen in the instantaneous PES. While the MEPs for aqueous DNA always pass via an intermediate G${}^{-}$C${}^{+}$ zwitterion, in both helicase scenarios, a small fraction of PES replicas exhibited MEPs passing through the alternate zwitterion G${}^{+}$C${}^{-}$. All seven replica MEPs proceeded via G${}^{-}$C${}^{+}$ for aqueous DNA while for the wild-type PcrA Helicase, only five of the seven replicas chose this path, and the N624A mutation showed this behaviour in three of the four replicas.

From the 2D-PES, we can determine the position of the proton corresponding to the G$^*$C$^*$ local minimum. To investigate the behaviour of the G$^*$C$^*$ state, we take the tunnelling product predicted by the MEP and place the protons back in the QM/MM ensemble snapshot from which the PES was generated (see Section 1.6 of the SI). All QM/MM trajectories resulted in the two protons transferring back to the canonical GC protonation state within 0.6 ps during these unbiased ensemble simulations (see Fig. S3 of the SI). The decay from the G$^*$C$^*$ tautomer to canonical GC was observed to take several pathways, one of which is illustrated on panel A of Fig. 4. While for some replicas, the MEP was found to go via the alternate zwitterion G${}^{+}$C${}^{-}$, no decay trajectories were found to take this path. However, in three out of seven trajectories within the wild-type PcrA helicase, the protons passed over or near the energetic maximum corresponding to a synchronous DPT. This behaviour was not observed in aqueous DNA.

Slocombe et al.^18,31^ have previously shown that imposing a separation on the DNA dimer stabilises the tautomeric product by increasing the reaction barrier of the minimum energy path (MEP). The primary action of the Helicase is to separate the strands of DNA, which will inevitably alter the DPT reaction profiles. During the umbrella sampling the GC dimer is compressed at the transition states, and both the canonical and tautomeric GC separations are higher for base pair N of the helicase than base pair N−1 and duplex DNA (see Section 2.3 and Fig. S6 of the SI). We have repeated our instantaneous MEP methodology for three steered MD snapshots at increasing separations (see Section 1.7 of the SI). Akin to the work by Slocombe et al.^18,31^, a steering force was applied to the backbone atoms of the GC dimer in the PcrA Helicase-DNA complex. Panels A and B of Fig. 5 show two example GC dimer configurations. We have verified that in the DNA-Helicase complex, the GC dimer opens asymmetrically (with the DG:N2-DC:O2 hydrogen-bond staying at near equilibrium) as reported in^18^. The instantaneous PES of the DPT was obtained, and the MEP was again determined with NEB. While the overall reaction asymmetry between canonical GC and the G$^*$C$^*$ tautomer was uncorrelated with the separation in this study, the two transition states and intermediate zwitterionic minimum in the triple well potential increased linearly with separation. Broadly these results indicate an agreement with previous mechanical studies which indicate a rapid reduction of proton transfer between canonical and tautomeric states as separation increases, which supports a “trapping” phenomenon where separation of a G$^*$C$^*$ base pair leads to greater stability of the tautomer. Our instantaneous PES study at increasing separation indicates that trapping is initially possible, but our US profiles do not exhibit a reverse reaction barrier, suggesting that such trapping would be short-lived as the system thermalises within 1 picosecond.

## Discussion

The US trajectories for the GC base pair at the opening of the ssDNA binding site of PcrA helicase show a considerable increase in the reaction free energy of both the G${}^{-}$C${}^{+}$ and G$^*$C$^*$ states. Furthermore, the presence of the helicase reduces the reverse barrier of the DPT reaction leading to a tautomer with lower stability than in isolated DNA. It is crucial to determine the stability of the G$^*$C$^*$ tautomeric base pair to clarify if they could survive the strand separation and potentially lead to point mutations. While previous models have anticipated the reaction barrier between canonical GC and G${}^{-}$C${}^{+}$ to increase with the mechanical action of a helicase enzyme, our results demonstrate for the first time the effect of a realistic model of the helicase-DNA complex on the stability of the tautomeric states. Additionally, we observe that asparagine N624 forms hydrogen bonds with a water molecule in the first solvation shell (see Fig. S8 of the SI), holding it in place close to the dissociating DNA and modifying the free energy landscape for proton transfer. This is further demonstrated by the increased density of water atoms surrounding the GC donor and acceptor atoms in base pair N of the helicase complex during classical MD, as seen in the Fig. S19 of the SI. Most notably, in base pair N of the PcrA complex the hydrogen acceptor O6 of the guanine has a 50% increased density around 1.7 Å when compared to both aqueous DNA and the N624A point mutated complex. This is consistent with the formation of a hydrogen bond in close proximity to a water molecule as we indeed observed in our QM/MM US trajectories, in good agreement with the results of Angiolari et al^20^. Angiolari et al.^20^ also suggested the presence of a water molecule in this configuration plays a role in destabilising the rare tautomeric and zwitterionic intermediate protonation states in GC. Substituting the asparagine with a hydrophobic alanine reduces this destabilising effect (see Fig. S8 of the SI). The remaining differences between duplex DNA and the alanine mutated helicase profile are attributed to the increase in base pair separation and altered chemical environment modelled by the electrostatic embedding of the QM/MM.

In duplex DNA, the GC $\rightarrow$ G$^*$C$^*$ tautomerisation takes place with a significant tautomeric population maintained at dynamic equilibrium^5,6,18^. However, the decreased stability of the G$^*$C$^*$ base pair at the active site of PcrA helicase suggests that one of the roles of the helicase is to protect against the formation of excess mutations. The stepping motor action of PcrA helicase, includes fast translocation dynamics (1 Å/ps) which are suspended for extended periods (10’s milliseconds) while a new ATP molecule diffuses into the binding pocket and is hydrolysed^18,24,26^. The most likely route for a point mutation to successfully pass the helicase enzyme is for the G$^*$C$^*$ base pairs population to be formed before base pair N in the DNA-helicase complex due to the significant reaction barrier and barrier-less reverse reaction in the helicase active site. The millisecond lag time between helicase’s translocation steps and the fractional picosecond lifetime of the tautomers suggests a further reduction in the tautomer population, and chemical equilibrium will have been reached.

Our novel instantaneous model for the proton transfer PES indicates that any such transfer reaction requires a significantly increased activation energy when compared to the equilibrium description (from US). It is conceivable that a rare tunnelling-ready state (TRS) may be populated via thermal fluctuations of the canonical configuration, in which the system is primed for transfer, akin to the mechanism reported in the Wobble-GT dimer in a polymerase palm domain^32^. A TRS would place the canonical system closer to the transition state and reduce the effective free energy barrier towards the zwitterion and tautomeric states. To promote tunnelling the protons would need to approach a configuration whose energy is degenerate with respect to the target well. In the PcrA helicase complex, our PESs indicate a GC system would need to gain $1.35\pm 0.13$ eV of thermal energy to be degenerate with the zwitterion G${}^{-}$C${}^{+}$. The intermediate G${}^{-}$C${}^{+}$ requires $0.43\pm 0.17$ eV to account for the reaction free energy to the G$^*$C$^*$ tautomeric well. Accounting for the low probability of thermally occupying a TRS in the canonical well (on the order $10^{-22}$), and then the zwitterionic well ($10^{-7}$), we expect the gain from thermally occupying states closer to the TS to be negligible.

However, while the tautomeric population is significantly diminished by the presence of the helicase, a small fraction of non-canonical GC dimers becomes trapped behind the rapidly increasing energy barrier when the hydrogen bonds begin dissociating. Consequently, the exact population of tautomers passing this process is determined by solving the microkinetics of the DPT process in a time-dependent PES, which we plan to study in closer detail in future studies. Here, we provide a semi-quantitative assessment of the probability of point mutations surviving the DNA splitting by assuming that the population ($1\times 10^{-8}$, see Table 1) in the rung down equilibrates in the helicase active site to a much lower concentration of $1\times 10^{-22}$. Determination of the tunnelling rate in a noisy environment and variable energy landscape poses a significant theoretical challenge. Simple corrections such as those proposed by Wigner^37^ and the WKB approximation^38^ are not strictly valid in this ’deep tunnelling’ regime and, as a result, would return a negligible tunnelling correction^5^. On the other hand, an accurate open quantum system description is required to capture the dynamics of the helicase environment. However, the magnitude of the free energy barriers reported here expects to diminish the tunnelling^6^.

Controlling the rate of mutations is one of the functions of a successful biological replication mechanism^29^, and whether or not the suppression of G$^*$C$^*$ mutagens by PcrA helicase is the result of a specific evolutionary pressure is an important question. Furthermore, the roles of well-conserved residues in the ssDNA binding site of PcrA helicase are known, and the asparagine N624 site is not shown to be vital to the stepping motor action of the enzyme as it in not part of the ssDNA binding domain^29^.

A survey of PcrA helicase sequences across 59 species was performed, exhibiting N624 in the majority (51%) of cases. Other amino acids present in this position are arginine (denoted R624 at 24%), glutamine (Q624 at 14%), and tyrosine (Y624 at 12%). R624 and Q624 strongly correlate with changes to other vicinal residues and occur within a multitude of different motifs between sites 622 and 626. These alternate sequences, as well as consideration of other helicase enzymes such as the 31 DNA helicases in the human genome^39^ and viral helicases^40–42^, might indeed be a fruitful route for future research, although we expect a similar increase in hydration around the hydrogen bonding site in most helicases leading to a destabilisation of rare protonation states.

Since the specific role of asparagine has been demonstrated in this work by the reduction in tautomer destabilisations following an alanine substitution, and N624 is not crucial for the successful function of PcrA helicase, it can be hypothesised that this specific amino acid was naturally selected to reduce mutations. In addition to demonstrating the need for further theoretical work on this area, we also provide an experimentally testable hypothesis that the N624A alanine substitution would reduce the rate of point mutation formation through the DPT.

While we have demonstrated the importance of modelling the explicit biological environments for understanding the role of proton transfer in DNA and the need for multiscale modelling to be included in future studies of GC tautomerism, to determine the precise interplay between a dynamically changing free energy landscape and the quantum delocalisation of GC’s protons. We have shown that the dynamics of DNA separation occur on the picosecond timescale and are sufficient to change the DPT reaction in duplex DNA radically^18^. The QM/MM MD decay trajectories give us critical insight into the short-lived nature of the metastable states, going beyond simple reaction kinetics. We observe G$^*$C$^*$ lifetimes on the order of tenths of picoseconds, suggesting that these processes proceed within the same timescale as strand separation and would need novel theoretical rate calculations before any definitive conclusions may be drawn.

## Methods

The duplex B-DNA structure was generated with Avogadro^43^. The atom and residue names were modified and suitable 3’ and 5’ termini were added using MolParse^44^. A modified and optimised version of the PcrA Helicase-DNA substrate complex PDB entry 3PJR,^23^ was obtained from Yu et al.^26^. The systems were solvated and neutralised in a cubic box of SPCE water with 1 nm clearance and sodium counter-ions.

Energy minimisation, molecular mechanics, and molecular dynamics were performed using Gromacs 2018^45^, with the March 2019 version of the CHARMM36 force field^46–48^. All calculations in Gromacs used the Verlet cutoff scheme applying 1 nm Coloumb and vdW radii, utilising fourth-order Particle Mesh Ewald long-range electrostatics, and cubic periodic boundary conditions.

Energy minimisation was performed on both these systems using a steepest descent algorithm until the maximum force did not exceed 12 kJ/mol/nm. Equilibration was performed in an NVT ensemble using a leap-frog integrator, timestep of 1 fs, and Nose-Hoover temperature coupling with time constant 0.2 ps and reference temperature 310 K, and three-dimensional periodic boundary conditions for a total of 3 ns.

Hybrid QM/MM calculations were performed with the Gromacs-CP2K distribution^45,49^. The GC nucleobase pair QM region was modelled with density functional theory, solved with CP2K^49^ with the BLYP exchange-correlation functional, DZVP-MOLOPT-GTH basis set, GTH-BLYP potential, electrostatic embedding, and the VDW3 dispersion correction. For QM/MM dynamics, Gromacs applied the leap-frog algorithm, with forces obtained from DFT in CP2K for the QM region and CHARMM36 elsewhere.

Umbrella Sampling was performed with Gromacs-CP2K at a CHARMM36/DFT/BLYP level of theory using four distance reaction coordinates describing the double proton transfer. Each umbrella sampling window applied a 20000 kJ/mol/nm$^2$ harmonic potential along the four reaction coordinates, and was simulated for at least 8 ps per replica per window. Depending on the level of equilibration, up to the first 2 ps of each simulation were discarded. The potentials of mean force (PMF) along the sampled reaction were obtained by the Weighted Histogram Analysis Method (WHAM) implemented in Gromacs with a tolerance of $1.0\times 10 ^{-6}$ and statistical variance was estimated with 100 bootstrapping samples. A logistic function was fit to the RMS difference between WHAM PMFs for the same system but varying sampling times to ensure even convergence between simulation systems (see Fig. S5 and Table S2 of the SI).

To determine the instantaneous double proton transfer potential energy surface, snapshots were taken from the QM/MM umbrella sampling simulations corresponding to the canonical GC. Each proton had its position interpolated between 0.9 and 2.5 Angstrom distance from its covalently bonded donor atom. Single point energies (SPEs) were calculated using the previously described electrostatically embedded QM/MM in CP2K with two levels of theory: identical to the umbrella sampling methodology with BLYP/DZVP-MOLOPT-GTH and B3LYP/DZVP-MOLOPT-GTH which also utilised the auxiliary density matrix method using the cFIT3 basis. The grid of SPEs was interpolated using the Clough-Tocher piecewise cubic, C1 smooth, curvature-minimizing interpolant in twodimensions implemented in SciPy.^50^ Within this surface local minima were determined using the BFGS optimiser implemented in ASE,^51^ and the minimum energy path connecting the canonical and tautomeric states was obtained using the nudged elastic band (NEB). To investigate the metastable nature of the G$^*$C$^*$ tautomer, post DPT product structures were generated from each instantaneous PES, and these were placed in unbiased 310 K NVT QM/MM MD simulations.

To obtain a strand separation trajectory in the DNA-Helicase complex a short (5ps) steered molecular dynamics simulation was performed on an equilibrated structure. A constant separating force (500 kJ/mol/nm$^2$) was applied to the DC:N1 and DG:N9 backbone atoms forcing them directly apart.

To determine the hydration of GC base pairs in various systems, additional molecular dynamics simulations (33 ns) were performed for three systems: aqueous DNA, the wild-type PcrA helicase-DNA complex, and the N624A point mutation of the same enzyme complex. From these trajectories, the hydrogen bonding geometries were analysed with MolParse^44^, and the rdf function was used to calculate the radial density of water around the hydrogen bonding sites of the GC dimer.

Further details regarding the computational simulation methodology can be found in section 1 of the supplementary information.

### Supplementary Information

Supplementary Information.

## Data Availability

The raw data used and analysed during the study are available from the corresponding author on request. In addition to further details provided in the Supplementary Information, input, parameter, and analysis files sufficient to reproduce the results published in this work are available on Github at: https://github.com/mwinokan/GC-tautomerism-in-PcrA-Helicase.
