# Supplementary material for: Multiscale simulations reveal the role of PcrA helicase in protecting against spontaneous point mutations in DNA
Source: Sci Rep. 2023 Dec 8;13:21749. doi: 10.1038/s41598-023-48119-z (PMC10709646; doi:10.1038/s41598-023-48119-z)
Supplement: Supplementary file 1 — Supplementary Information. [file 41598_2023_48119_MOESM1_ESM.pdf]

Supporting Information for: Multiscale simulations reveal the role of PcrA helicase in protecting against spontaneous point mutations in DNA

Max Winokan[a,1], **Louie Slocombe**[b,2], **Jim Al-Khalili**[c,3], **and Marco Sacchi**[b,4]

[a]Leverhulme Quantum Biology Doctoral Training Centre, University of Surrey, Guildford, GU2 7XH, UK.
[b]Department of Chemistry, University of Surrey, Guildford, GU2 7XH, UK.
[c]School of Mathematics and Physics, University of Surrey, Guildford, GU2 7XH, UK.
[1]E-mail: m.winokan@surrey.ac.uk
[2]E-mail: louie.slocombe@surrey.ac.uk
[3]E-mail: j.al-khalili@surrey.ac.uk
[4]E-mail: m.sacchi@surrey.ac.uk

June 2023

This document contains the supplementary information for the article entitled: "Multiscale simulations reveal the role of PcrA helicase in protecting against proton transfer in DNA". In addition to the computational methodology (Section 1), additional simulation details (Section 2), and additional simulation results (Section 3), input, parameter, and analysis files sufficient to reproduce the results published in this work are available on Github at `https://github.com/mwinokan/GC-tautomerism-in-PcrA-Helicase`.

1 Computational Methodology

This section describes the computational methodology used in this work. Broadly, the computational research undertaken in this work involved the mapping of double proton transfer within Guanine-Cytosine in an aqueous DNA duplex (as a control), and in the bacterial PcrA Helicase-DNA complex.

1.1 System Preparation

The duplex B-DNA structure was generated with Avogadro(1) using the sequence shown in Table S1. The atom and residue names were modified for use with the CHARMM36(2–4) force field and the Gromacs(5) software and suitable 3' and 5' termini were added using MolParse(6).

A modified and optimised version of the PcrA Helicase-DNA substrate complex PDB entry 3PJR,(7) was obtained from Yu et al. (8).

With tools included with Gromacs, topologies were generated for these systems using the March 2019 CHARMM36 force field. The systems were solvated in a cubic box of SPCE water ensuring at least 1 nanometre of clearance between any non-solvent atom and the edge of the box. The system was neutralised with the addition of sodium ions.

1.2 Molecular Dynamics Equilibration

All energy minimisation, molecular mechanics, and molecular dynamics were performed using Gromacs 2018(5), with the March 2019 version of the CHARMM36 force field, and SPCE water.

Prior to any dynamics energy minimisation was performed on both these systems using a steepest descent algorithm until the maximum force did not exceed 12 kJ/mol/nm. The molecular dynamics parameters for the geometry optimisation can be found in gromacs_parameters/minim_12.mdp.

Each of these systems was placed simulated in an NVT ensemble using a leap-frog integrator, timestep of 1 fs, and Nose-Hoover temperature coupling with time constant 0.2 ps and reference temperature 310 K, and three-dimensional periodic boundary conditions for a total of 3 ns. Within this time the systems were verified to have been equilibrated as the temperatures and RMSD stabilised. An example molecular dynamics parameter file for the molecular dynamics equilibration can be found in gromacs_parameters/eq500ps_2CX.mdp.

1.3 Hybrid Quantum Mechanics / Molecular Mechanics

Hybrid QM/MM calculations were performed with the Gromacs-CP2K distribution(5, 9). The nucleobases of the relevant GC base pair were defined as the QM region, whose electronic structure would be solved CP2K(9) using density functional theory with the BLYP exchange correlation functional, DZVP-MOLOPT-GTH basis set, GTH-BLYP potential, electrostatic (point charge) embedding, and the VDW3 dispersion correction. For QM/MM dynamics, Gromacs applied the leap-frog algorithm, with forces obtained from DFT in CP2K for the QM region and CHARMM36 elsewhere.

1.4 Umbrella Sampling

Umbrella Sampling was performed with Gromacs-CP2K at a CHARMM36/DFT/BLYP level of theory using four distance reaction coordinates describing the double proton transfer as illustrated in Figure S1. Each umbrella sampling window applied a 20000 kJ/mol/nm^2 harmonic potential along the four reaction coordinates, and was simulated for at least 8ps per replica per window. Depending on the level of equilibration, up to the first 2 ps of each simulation were discarded. An example for the steered molecular dynamics parameter input file for Gromacs can be found in gromacs_parameters/umb_8ps_f20k_2CM_BLYP_notraj.mdp, and a corresponding example CP2K input file in cp2k_parameters/HS41R1_BLYP_VDW_w008.inp.

The double proton transfer pathways that were sampled are illustrated in Figure S2. The concerted/synchronous DPT is a simple linear interpolation between the canonical GC and the G*C* tautomer. The asynchronous pathway was taken from the equilibrium distance GC dimer ML-NEB pathways reported by Slocombe et al.(10)

The potentials of mean force (PMF) along the sampled reaction were obtained by the Weighted Histogram Analysis Method (WHAM) implemented in Gromacs with a tolerance of 1.0×10^{-6} and statistical variance was estimated with 100 bootstrapping samples. The four reaction coordinates were projected onto one dimension in an average relative change from the canonical minimum, which is the default for Gromacs' WHAM. The reaction coordinate z is thus defined as:

$$z = \frac{(\mathrm{RC1} - \mathrm{RC1}_0) + (\mathrm{RC3} - \mathrm{RC3}_0) - (\mathrm{RC2} - \mathrm{RC2}_0) - (\mathrm{RC4} - \mathrm{RC4}_0)}{4} + \frac{\mathrm{RC1}_0 + \mathrm{RC3}_0}{2} \tag{1}$$

Where $\mathrm{RC1}_0$ is the canonical reference point for RC1, etc.

The reaction asymmetries obtained from PMFs were used to calculate the equilibrium constants (K) using Equation 2. Where ΔG is a reaction free energy, R is the gas constant, and T is temperature (310K in this work).

$$\ln K = -\frac{\Delta G}{RT} \tag{2}$$

1.5 Instantaneous Transfer Surface

To determine the instantaneous double proton transfer potential energy surface, snapshots were taken from the QM/MM umbrella sampling simulations corresponding to the canonical GC. 256 replica structures were created using Python and MolParse(6) mapping the two dimensional transfer landscape of the two protons. Each proton had its position interpolated between 0.9 and 2.5 Angstrom distance from its covalently bonded donor atom. The python code in analysis_code/grid.py was used to generate the array of input structures.

For each of these grid structures a single point energy (SPE) was calculated using the previously described electrostatically embedded QM/MM in CP2K with two levels of theory: identical to the umbrella sampling methodology with BLYP/DZVP-MOLOPT-GTH and B3LYP/DZVP-MOLOPT-GTH which also utilised the auxillary density matrix method using the cFIT3 basis. For each SPE calculation a new CP2K input was constructed using the following parameter templates: BLYP cp2k_parameters/template_BLYP.inp and B3LYP cp2k_parameters/template_B3LYP_new.inp.

The grid of SPEs was interpolated using the CloughTocher piecewise cubic, C1 smooth, curvature-minimizing interpolant in two-dimensions inplemented in SciPy.(11) Within this surface local minima were determined using the BFGS optimiser implemented in ASE,(12) and the minimum energy path connecting the canonical and tautomeric states was obtained using the nudged elastic band (NEB) algorithm also implemented in ASE. All of this functionality was achieved the analysis code in analysis_code/plot_grid.py

1.6 G*C* Ensemble decay

To investigate the metastable nature of the G*C* tautomer, post DPT product structures were generated from each instantaneous PES, and these were placed in unbiased 310K NVT QM/MM MD simulations, the parameters for which can be found in gromacs_parameters/md5ps_qmmm_vel.mdp. The velocities were taken from the original US snapshot used to generate the surface.

To quantitatively compare the decays the time at which the system leaves the local potential energy minima has been estimated by considering the point where the two reaction coordinates cross over. For example, in the DC:N4-DC:H41 – DG:O6 transfer described by RC1 and RC3 (see Subsection 1.4) we consider the proton to be in the canonical well as long as RC1 < RC3. Additionally once the vector between the canonical minima and [RC1,RC3] is within 0.1 Angstrom, we consider the system to have 'arrived' at the canonical GC state. These lifetime results are shown in Figure S3.

1.7 Instantaneous PES at non-equilibrium separation

To obtain a strand separation trajectory in the DNA-Helicase complex a short (5ps) steered molecular dynamics simulation was performed on an equilibrated structure. A constant separating force (500 kJ/mol/nm^2) was applied to the DC:N1 and DG:N9 backbone atoms forcing them directly apart. See gromacs_parameters/pull_v1.mdp for exact simulation parameters.

1.8 Mutations of the PcrA Helicase sequence

Sequences for PcrA Helicase from 59 species were analysed around site 624 which is an asparagine (N624) in 51% of cases, other minor populations of 624 include arginine (R624, 24%), glutamine (Q624, 14%), and tyrosine (Y624, 12%). Table S4 shows the statistics of mutations in the sites 622 to 626. When considering vicinal residues to N624 multiple motifs appear. Motifs with N624 remain the most common, with FG**N**IQ, and FG**N**IH making up the 51% majority. When considering sites 622 to 626, a tyrosine at 624 (Y624) is always part of a FG**Y**TN motif (12% frequency), while sequences containing glutamine Q624 and arginine R624 are more diverse, i.e. the vicinal positions 622, 623, 625, and 626 are not strongly correlated to the amino acid in 624.

1.9 Additional Molecular Dynamics trajectories for aqueous DNA and the PcrA Helicase-DNA complex

In addition to the 3 ns of equilibration time, three systems were simulated across a further 30 ns each to observe slower dynamics. The same Molecular Dynamics (MD) parameters were used as described in Subsection 1.2.

2 Additional Simulation Details

This section provides further details regarding the validation of the methods described in Section 1.

2.1 QM/MM level of theory

The exchange correlation functional BLYP with and without VDW3 dispersion corrections was compared to PBE and the semi-empirical PM6 for the concerted DPT in aqueous DNA, and the PMFs are shown in Figure S4. PM6 provided the greatest statistical uncertainty between all tested levels of theory and was thus not a suitable description of this reaction. Between BLYP, PBE, and BLYP+VdW3 there are small differences in the produced concerted double proton transfer barriers. BLYP+VdW3 was chosen for the umbrella sampling due to its faster sampling convergence. See Subsection 2.2.

2.2 Sampling convergence

To ensure that the potentials of mean force produced from the umbrella sampling simulations have converged the RMS difference between the reaction profiles was calculated for a given sampling time. For the chosen level of theory (BLYP VDW) a convergence is reached within 500ps of umbrella sampling, see Figure S5. Table S2 shows the total sampling time for each umbrella sampling PMF obtained in this work. Despite the computational expense of QM/MM umbrella sampling, we have ensured that the asynchronous pathway PMF for each studied system has converged to above 95%.

2.3 GC dimer separation during umbrella sampling simulations

During the umbrella sampling reactions the average donor-acceptor distances of the hydrogen bonds involved in proton transfer varied significantly and are reported in Figure S6. Mathematically the separation can be described as $(RC1 + RC2 + RC3 + RC4)/4$. We report a compression of the dimer during the sampled reaction, as previously observed by Slocombe *et al.* in (13) and (10). For the concerted double proton transfer a single compression is observed, corresponding to the single transition state, except the Helicase N-1 case which has a compressed tautomeric state. For the asynchronous pathway we observe two compression events corresponding to the two transition states resisting each proton transfer, again, the N-1 case was an outlier and did not exhibit this compression.

This compression shows the high energetic cost of the proton transfer is alleviated by molecular degrees of freedom, not directly involved in the proton transfer. This motivates the need for a description of the proton transfer without such atomic rearrangement, especially if the proton transfer is known to occur near-instantaneously due to quantum tunnelling.

3 Additional Simulation Results

This section provides additional results to support discussions in the main text.

3.1 Umbrella sampling results for the synchronous DPT

While the asynchronous double proton transfer (DPT) was found to be energetically preferred with both umbrella sampling (US) and the instantaneous minimum energy path (MEP), we also report the synchronous proton transfer in Table 1 of the main article. The corresponding potentials of mean force (PMF's) are shown in S7.

3.2 Conformational behaviour of aqueous DNA and the PcrA-Helicase in longer molecular dynamics trajectories

Aqueous DNA, the wild type PcrA Helicase-DNA complex (N624), and the point mutation N624A were simulated in longer 33ns NVT MD (see Subsection 1.9).

For Aqueous DNA, an overview of the dynamics is shown in Figure S15, which shows significant fraying at the ends of the duplex, while more stability is maintained in the middle. Figure S18 shows the donor-acceptor distance for the two hydrogen bonds involved in the DPT from these extended MD trajectories. The distribution for both bonds - DGO6-DCN4 (proton 1's donor and acceptor, and sum of RC1 and RC2) and DGN1-DCN3 (proton 2's donor and acceptor, and sum of RC3 and RC4) - centre on a donor-acceptor distance of 3 Å.

For the wild-type PcrA Helicase-DNA complex with asparagine N624, the conformations present in the extended MD are summarised in Figure S16, in which panel A shows significant fluctuations in the single-stranded DNA not passing through the enzyme, but relative stability across the rest of the complex. For base pair N, Panel B shows the conformation used for umbrella sampling and corresponds to the 3 Å peak of the donor-acceptor distance distribution shown in Figure S18. Panel C shows the a common alternative conformation corresponding to the more separated peak around 5.7 Å. While the GC base pair N-1 did open up slightly further than the aqueous DNA reference (deduced from the longer tail in the donor-acceptor distance distribution), the distribution still peaks at the equilibrium value (3 Å).

For the PcrA-DNA complex with the N624A point mutation, the smaller sidechain of alanine compared to asparagine has allows for increased mobility of the single-stranded DNA and a different alternative conformation is formed (see panel C of Figure S17). In this alternative conformation, DG662 and DC701 (base pair N) are no longer the last in the duplex as the DT663-DA702 (pair N+1) can reform. It is also observed that the alternative conformation does not stretch the base pair N (see the donor-acceptor distance in Figure S18), as most of the distribution is still centred around 3 Å. However, likely due to the increased mobility of the ssDNA, base pair N is opens more frequently during the simulations than in aqueous DNA, but not as often as in complex with wild-type PcrA.

3.3 Instantaneous DPT transfer surfaces

The individual instantaneous DPT transfer surfaces described in the main text are shown in Figures S9, S11, and S13 for duplex DNA, wild type PcrA Helicase (base pair N), and for helicase with the N624A mutation, respectively. The minimum energy paths through the surfaces are shown in Figures S10, S12, and S14, for the same three systems.

Table S1. Sequence of duplex DNA used for simulations.

Chain	1	2	3	4	5	6	7	8	9	10	11	12	13	14	15	16	17	18	19	20	21	22	23	24
1	C_5	A	G	T	G	C	A	G	T	G	C	T	C	G	T	T	T	T	T	T	T	T	T	T_3
2	G_3	T	C	A	C	G	T	C	A	C	G	A	G	C	A	A	A	A	A	A	A	A	A	A_5

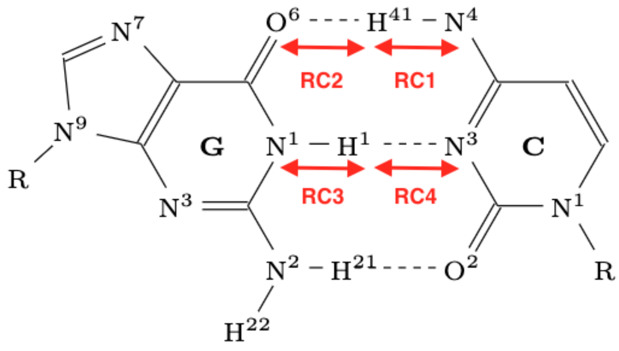

Fig. S1. Reaction coordinates used to map the double proton transfer

From these figures we can determine that the instantaneous PES and MEP through it is highly sensitive to conformational differences between snapshots. The locations and energies of the PES minimas are summarised in Table S3. The locations of the non-canonical minima vary between replicas of the same system, as the length of the hydrogen bonds and separation of the GC dimer fluctuate during the MD and US (see 2.3). Similarly, conformational differences lead to large energetic differences between the non-canonical minima between replicas, by as much as 28% relative standard deviation.

Table S2. QM/MM umbrella sampling: total simulation time for free energy calculations

System	Path	Base Pair	Site 624	Sampling [ps]
Aqueous Duplex DNA	Concerted	-	-	436.0
Aqueous Duplex DNA	Asynchronous	-	-	789.0
PcrA Helicase-DNA Complex	Concerted	N	N	408.0
PcrA Helicase-DNA Complex	Asynchronous	N	N	1008.7
PcrA Helicase-DNA Complex	Concerted	N-1	N	288.0
PcrA Helicase-DNA Complex	Asynchronous	N-1	N	480.0
PcrA Helicase-DNA Complex	Asynchronous	N	A	797.8

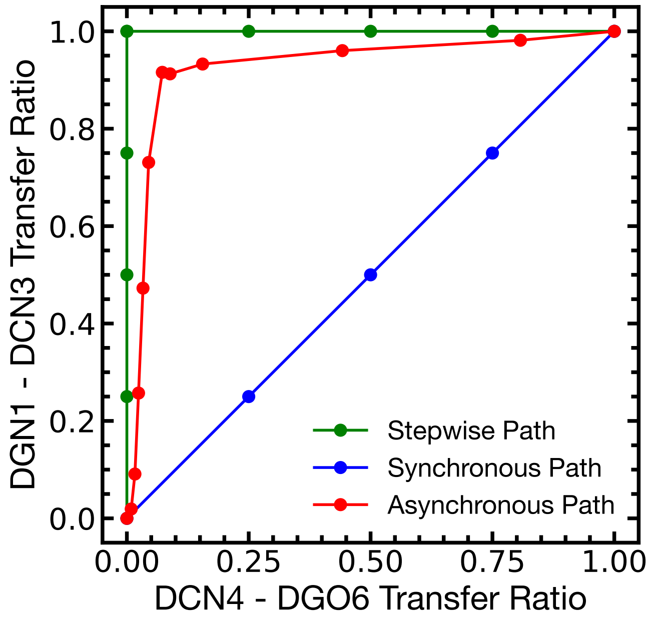

Fig. S2. Reaction paths used in Umbrella Sampling

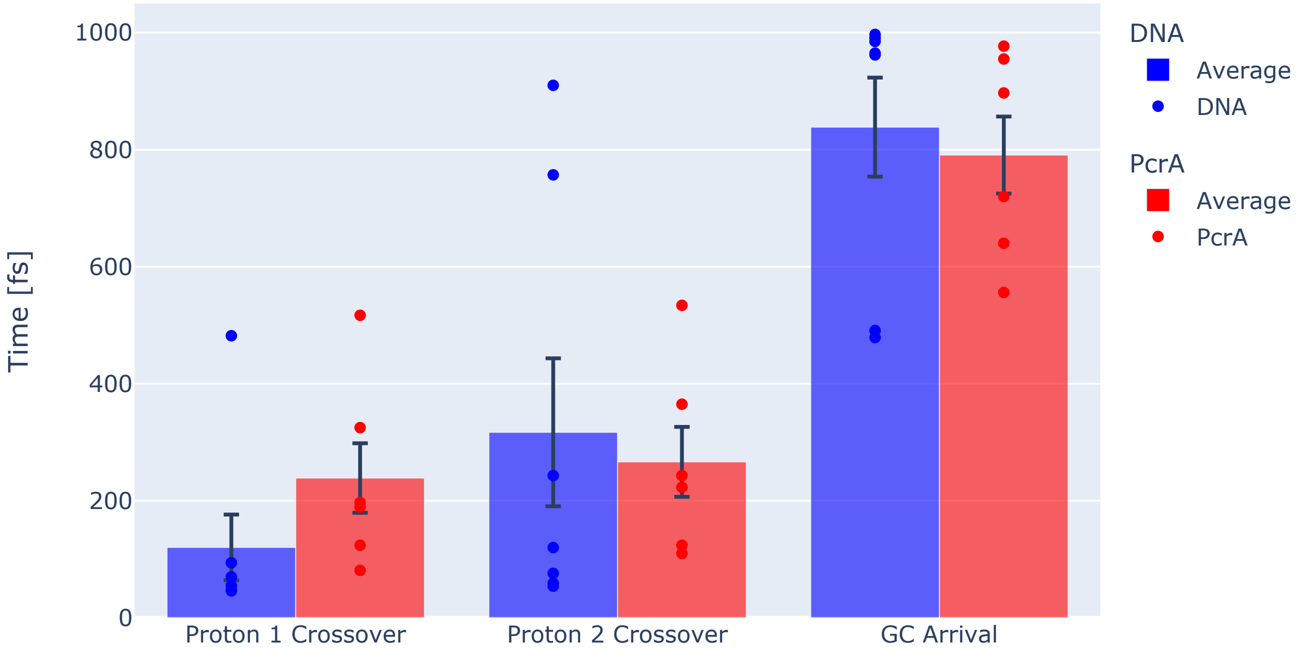

Fig. S3. Lifetime estimation for the G*C* tautomer in duplex DNA and the PcrA Helicase-DNA complex.

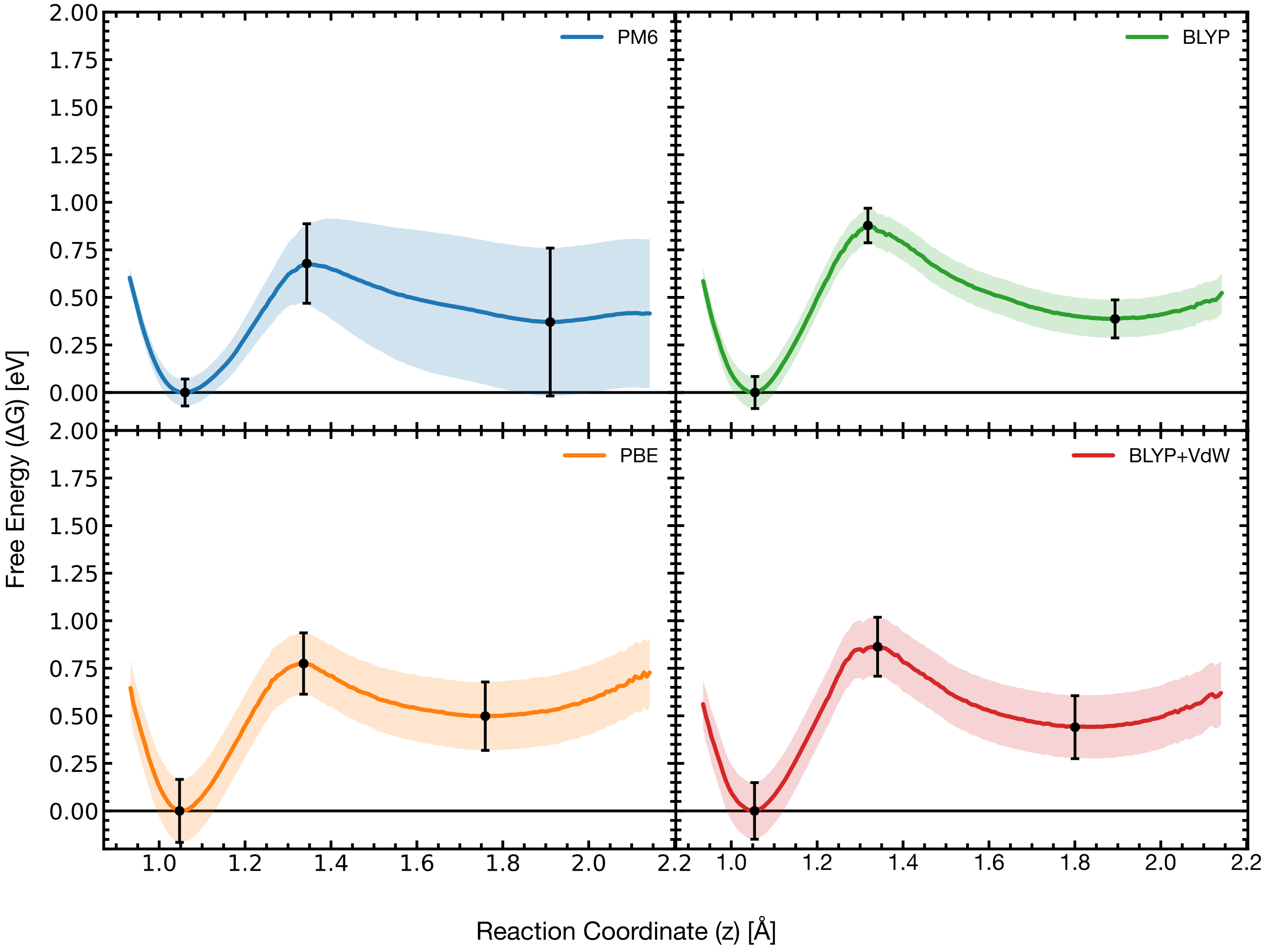

Fig. S4. Umbrella sampling potentials of mean force for different levels of QM theory.

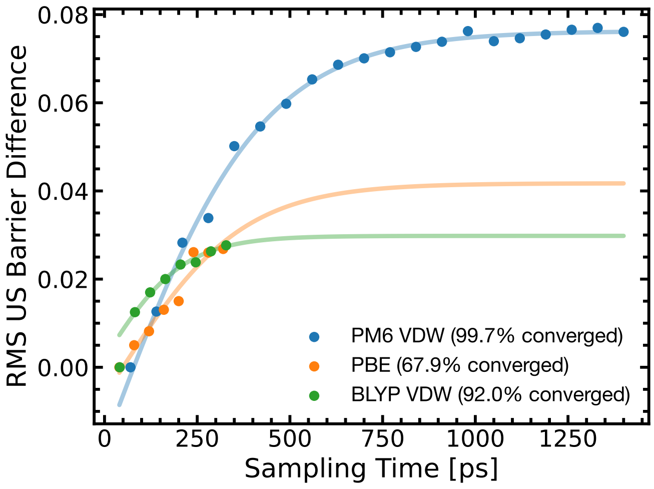

Fig. S5. Convergence of the umbrella sampling reaction profiles with increasing sampling time

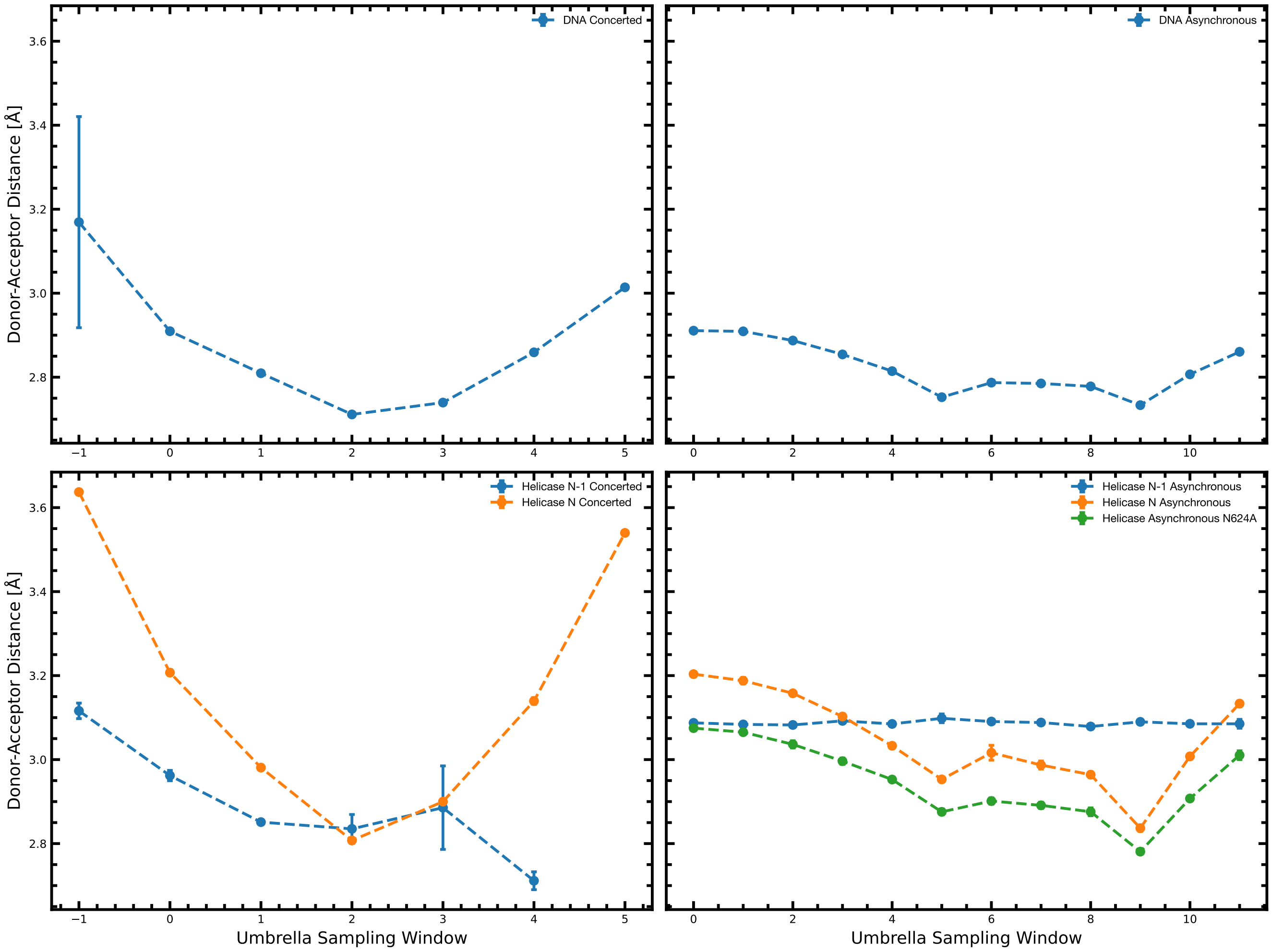

Fig. S6. Average GC dimer hydrogen bond donor-acceptor distance during umbrella sampling.

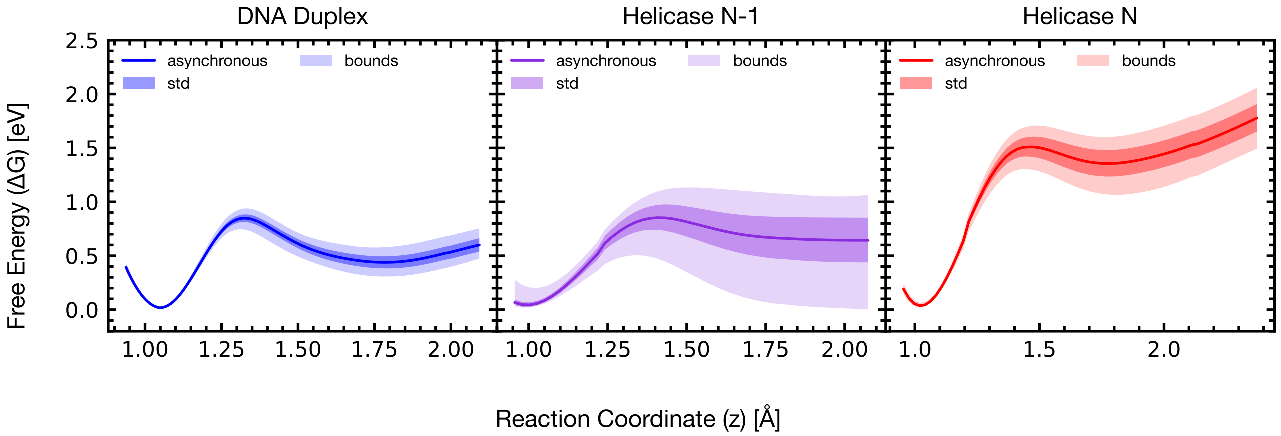

Fig. S7. Potentials of mean force (PMF) for the synchronous double proton transfer in guanine-cytosine obtained from QM/MM Umbrella Sampling (US) simulations for Aqueous Duplex DNA (A) and two base pairs in the wild type PcrA Helicase-DNA Complex, pair N-1 (B) and pair N (C).

Table S3. Properties of energetic minima in instantaneous potential energy surfaces. RC1 and RC3 are the donor-hydrogen distance for each hydrogen bond involved in the DPT (see Figure S1), and ΔE is the potential energy relative to the canonical (GC) minimum.

Duplex DNA	GC			$G^{-}C^{+}$			$G^{+}C^{-}$			$G^{*}C^{*}$		
Property	RC1(Å)	RC3(Å)	ΔE(eV)	RC1(Å)	RC3(Å)	ΔE(eV)	RC1(Å)	RC3(Å)	ΔE(eV)	RC1(Å)	RC3(Å)	ΔE(eV)
mid_L01	1.028	1.048	0.000	1.044	1.779	0.650	-	-	-	1.922	1.830	1.303
mid_L02	1.030	1.036	0.000	1.050	2.002	0.953	-	-	-	1.751	2.047	1.461
mid_L03	1.033	1.048	0.000	1.054	1.941	0.482	-	-	-	1.812	1.966	0.918
mid_L05	1.046	1.061	0.000	1.056	1.511	0.426	-	-	-	1.735	1.637	0.689
L03_c4	1.018	1.053	0.000	1.046	1.769	0.697	-	-	-	1.764	1.825	1.344
L05	1.020	1.060	0.000	1.043	1.773	0.456	1.895	1.083	1.618	1.957	1.827	1.032
L11	1.029	1.047	0.000	1.053	1.835	0.592	-	-	-	1.740	1.876	1.168
avg.	1.029	1.050	0.000	1.049	1.801	0.608	1.895	1.083	1.618	1.811	1.858	1.131
std.	0.008	0.008	0.000	0.005	0.145	0.170	0.000	0.000	0.000	0.085	0.119	0.249
std. (%)	0.8	0.8	0.0	0.5	8.1	27.9	0.0	0.0	0.0	4.7	6.4	22.1
PcrA (N624)	GC			$G^{-}C^{+}$			$G^{+}C^{-}$			$G^{*}C^{*}$		
Property	RC1(Å)	RC3(Å)	ΔE(eV)	RC1(Å)	RC3(Å)	ΔE(eV)	RC1(Å)	RC3(Å)	ΔE(eV)	RC1(Å)	RC3(Å)	ΔE(eV)
HL01	1.016	1.041	0.000	1.038	1.899	1.500	2.230	1.059	1.964	2.262	1.997	1.735
HL02	1.003	1.046	0.000	1.035	1.984	1.204	2.237	1.062	2.316	2.279	2.041	1.674
HL03	1.018	1.046	0.000	-	-	-	2.113	1.074	1.794	2.161	1.827	1.493
HL04	1.013	1.038	0.000	1.031	1.855	1.685	2.182	1.052	2.267	2.224	1.957	2.280
HL05	1.013	1.038	0.000	1.032	1.864	1.391	2.188	1.058	2.086	2.222	1.936	1.812
HL06	1.041	1.019	0.000	1.041	1.874	1.817	2.273	1.041	1.581	2.282	1.985	1.648
grid_new	1.017	1.048	0.000	1.033	1.984	1.445	2.286	1.055	2.572	2.322	2.081	2.214
avg.	1.017	1.039	0.000	1.035	1.910	1.507	2.216	1.057	2.083	2.250	1.975	1.837
std.	0.011	0.009	0.000	0.004	0.054	0.199	0.055	0.009	0.311	0.049	0.075	0.275
std. (%)	1.0	0.9	0.0	0.4	2.8	13.2	2.5	0.9	14.9	2.2	3.8	15.0
PcrA (N624A)	GC			$G^{-}C^{+}$			$G^{+}C^{-}$			$G^{*}C^{*}$		
Property	RC1(Å)	RC3(Å)	ΔE(eV)	RC1(Å)	RC3(Å)	ΔE(eV)	RC1(Å)	RC3(Å)	ΔE(eV)	RC1(Å)	RC3(Å)	ΔE(eV)
N624A11	1.014	1.043	0.000	1.037	1.897	1.240	2.129	1.066	2.259	2.196	1.970	1.809
N624A12	1.019	1.046	0.000	1.047	1.822	1.030	1.862	1.067	1.622	1.935	1.890	1.368
N624A13	1.016	1.039	0.000	-	-	-	2.286	1.053	2.021	2.303	1.907	1.883
N624A14	1.013	1.041	0.000	1.034	1.889	1.229	2.280	1.056	2.425	2.291	1.962	1.886
avg.	1.015	1.042	0.000	1.039	1.869	1.166	2.139	1.060	2.082	2.181	1.932	1.737
std.	0.002	0.003	0.000	0.006	0.033	0.096	0.172	0.006	0.302	0.148	0.035	0.215
std. (%)	0.2	0.3	0.0	0.5	1.8	8.3	8.0	0.6	14.5	6.8	1.8	12.4

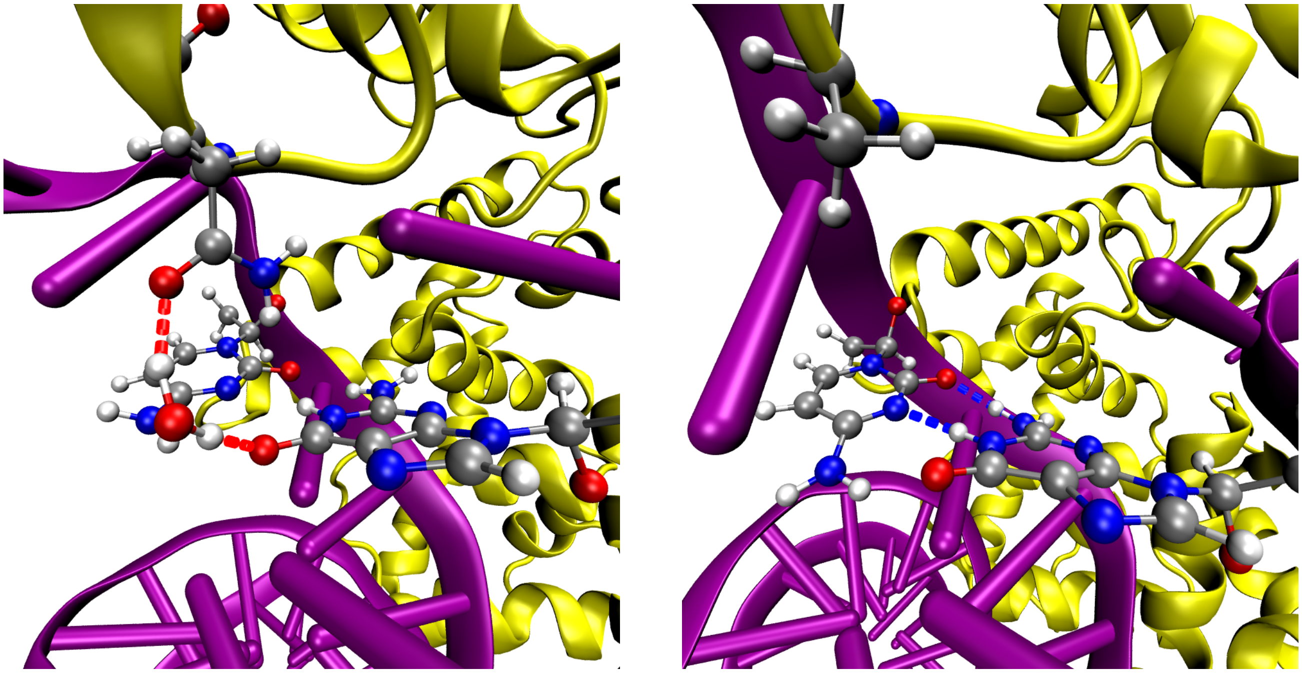

Fig. S8. Archetypical conformations of the PcrA Helicase-DNA duplex with the wild type sequence and N624A point mutation. The base pair N GC dimer and amino acid in site 624 are shown in CPK representation.

Table S4. Amino acid residues in sites 622-626 across 59 species of PcrA Helicase. Asterisks (*) are used as a wildcard denoting any amino acid. N is the number of occurences of the given motif, also given as a percentage in parentheses.

622	623	624	625	626	N (%)
*	*	N	*	*	30 (51%)
*	*	R	*	*	14 (24%)
*	*	Q	*	*	8 (14%)
*	*	Y	*	*	7 (12%)
*	G	N	I	*	30 (51%)
*	G	R	T	*	14 (24%)
*	G	Q	T	*	8 (14%)
*	G	Y	T	*	7 (12%)
F	G	N	I	Q	24 (41%)
F	G	Q	T	H	7 (12%)
F	G	Y	T	N	7 (12%)
F	G	N	I	H	6 (10%)
F	G	R	T	N	5 (8%)
Y	G	R	T	N	2 (3%)
F	G	R	T	G	2 (3%)
F	G	R	T	M	1 (2%)
F	G	R	T	A	1 (2%)
F	G	R	T	T	1 (2%)
F	G	R	T	S	1 (2%)
F	G	Q	T	N	1 (2%)
Y	G	R	T	E	1 (2%)

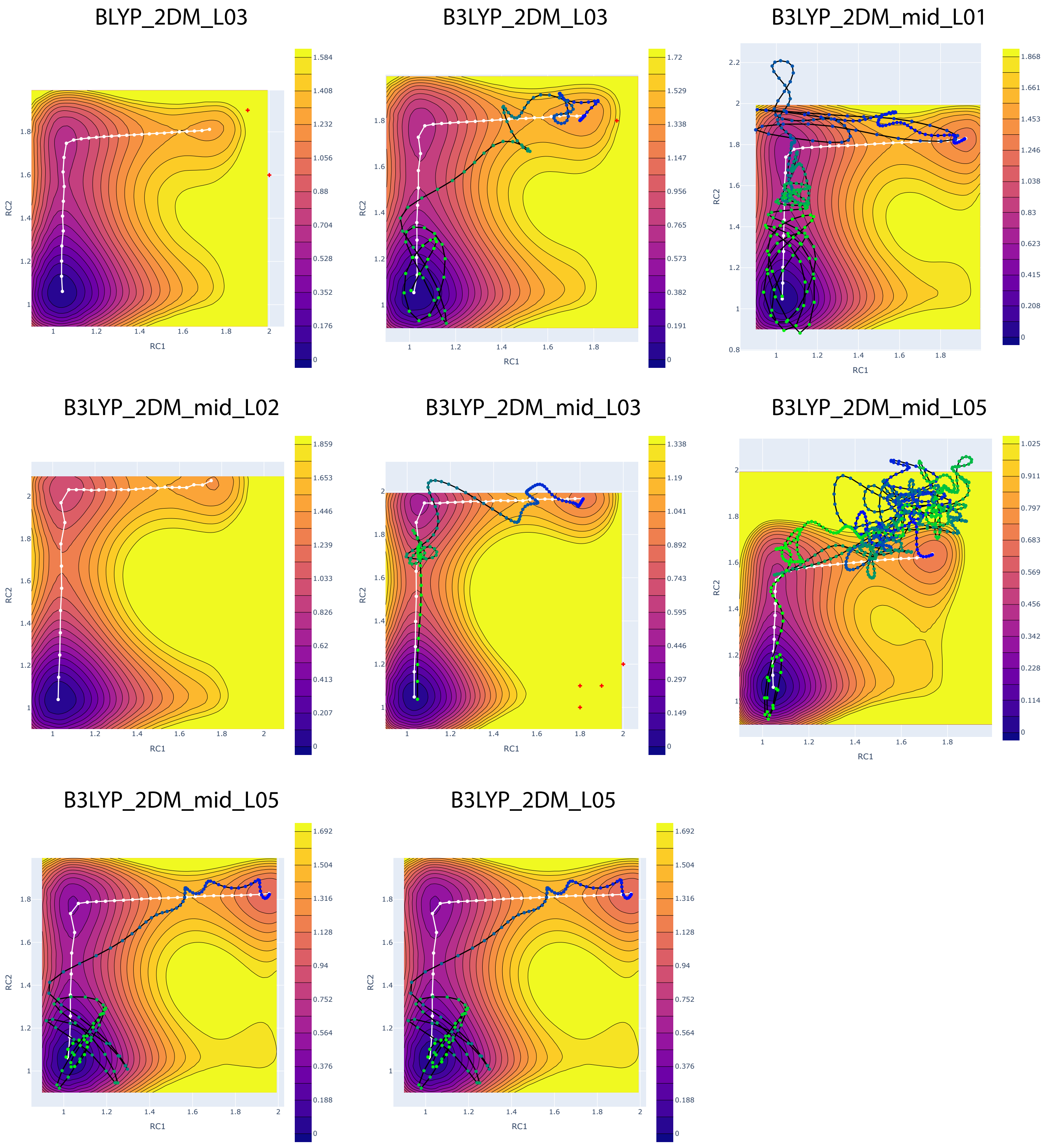

Fig. S9. Instantaneous DPT PES for duplex DNA. Contoured heatmap illustrating the DPT's instantaneous energy surface, the minimum energy pathway within this landscape (white line with circular dots), and the decay path from G*C* to GC (black line with multicoloured circles). For the decay path, the dots are coloured according to the simulation time, with blue corresponding to the start of the simulation and green at the end of the decay.

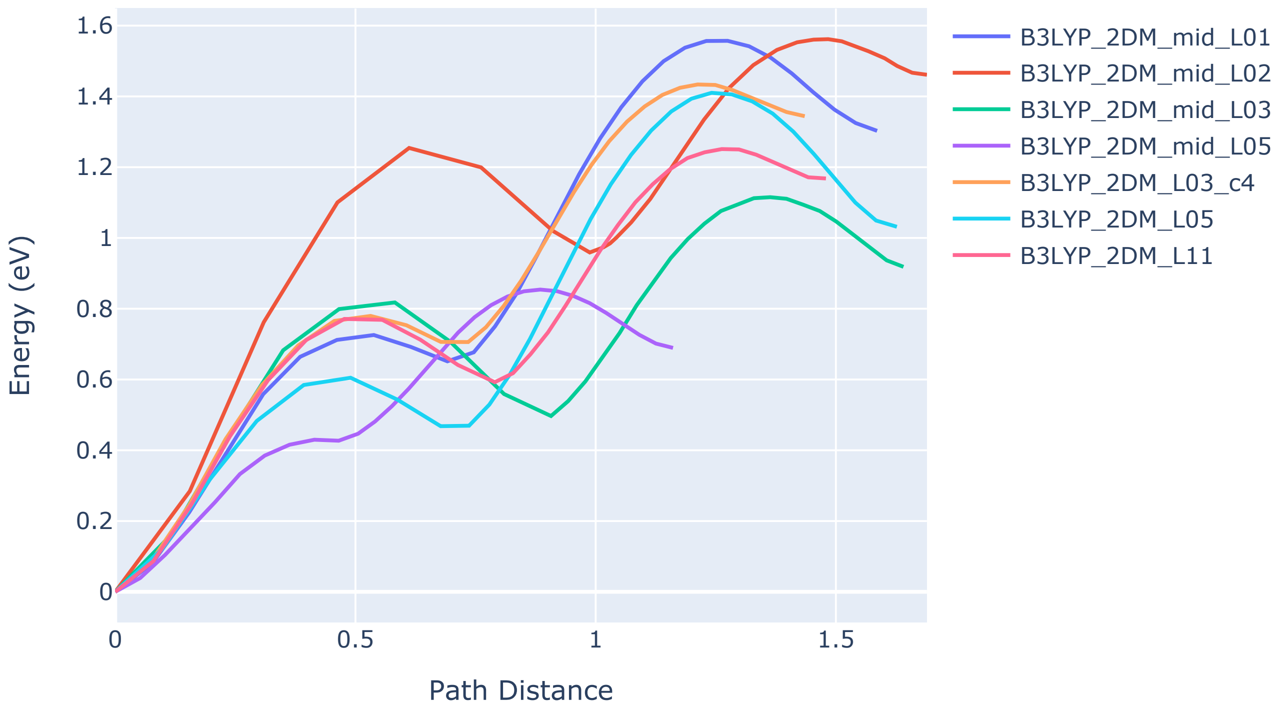

Fig. S10. Minimum energy paths through the instantaneous DPT PES for duplex DNA.

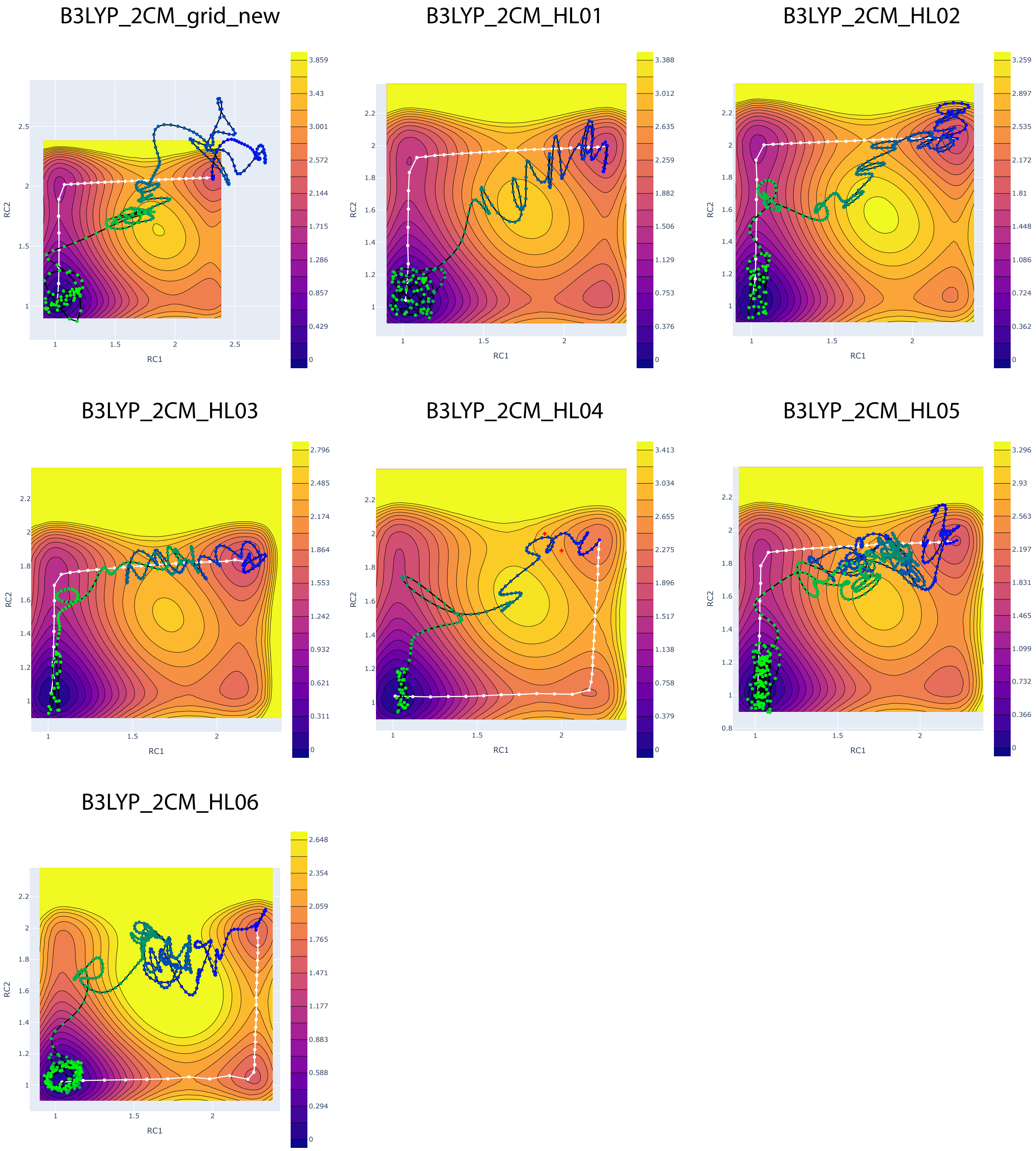

Fig. S11. Instantaneous DPT PES for the PcrA Helicase-DNA complex (base pair N). Contoured heatmap illustrating the DPT's instantaneous energy surface, the minimum energy pathway within this landscape (white line with circular dots), and the decay path from G*C* to GC (black line with multicoloured circles). For the decay path, the dots are coloured according to the simulation time, with blue corresponding to the start of the simulation and green at the end of the decay.

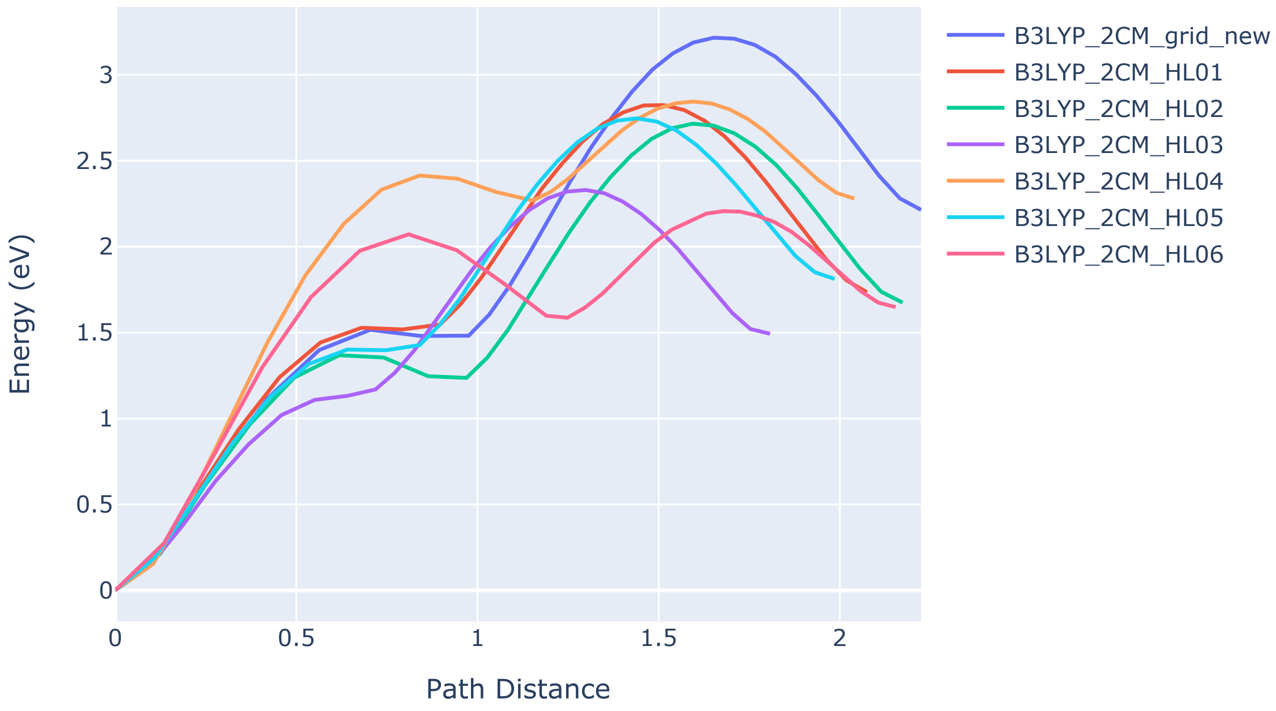

Fig. S12. Minimum energy paths through the instantaneous DPT PES for the PcrA Helicase-DNA complex (base pair N).

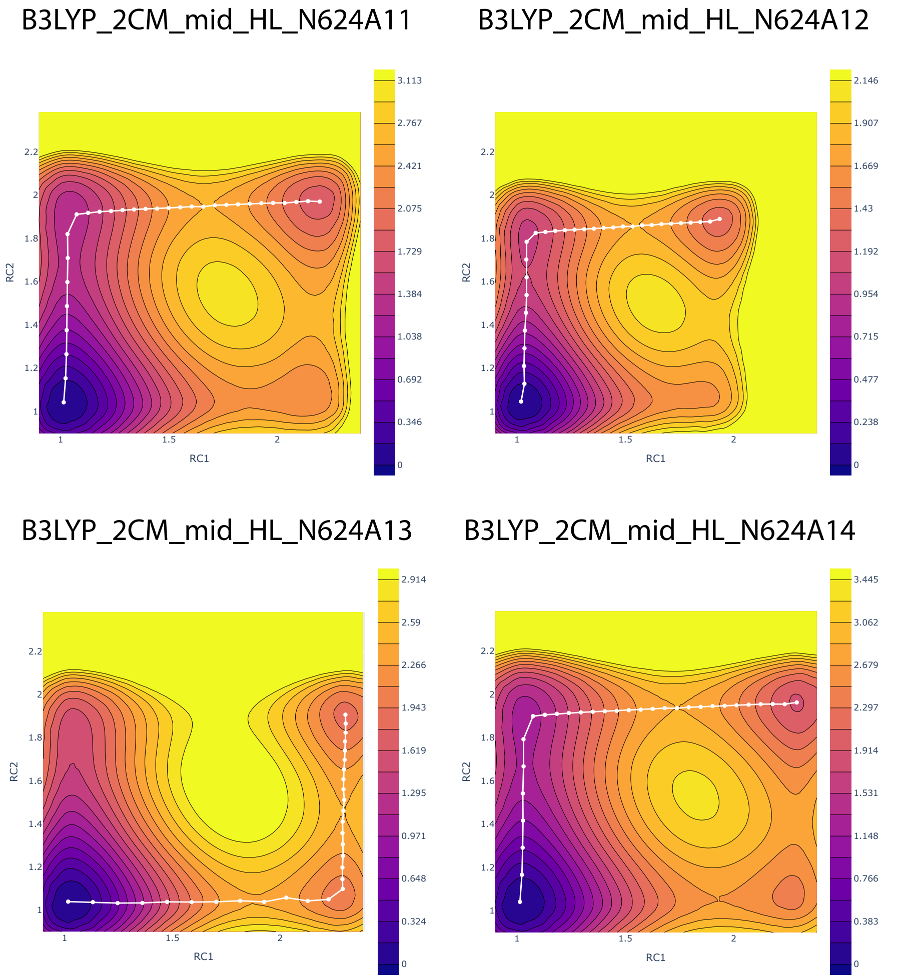

Fig. S13. Instantaneous DPT PES for the PcrA Helicase-DNA complex (base pair N) with the N624A point mutation. Contoured heatmap illustrating the DPT's instantaneous energy surface, the minimum energy pathway within this landscape (white line with circular dots).

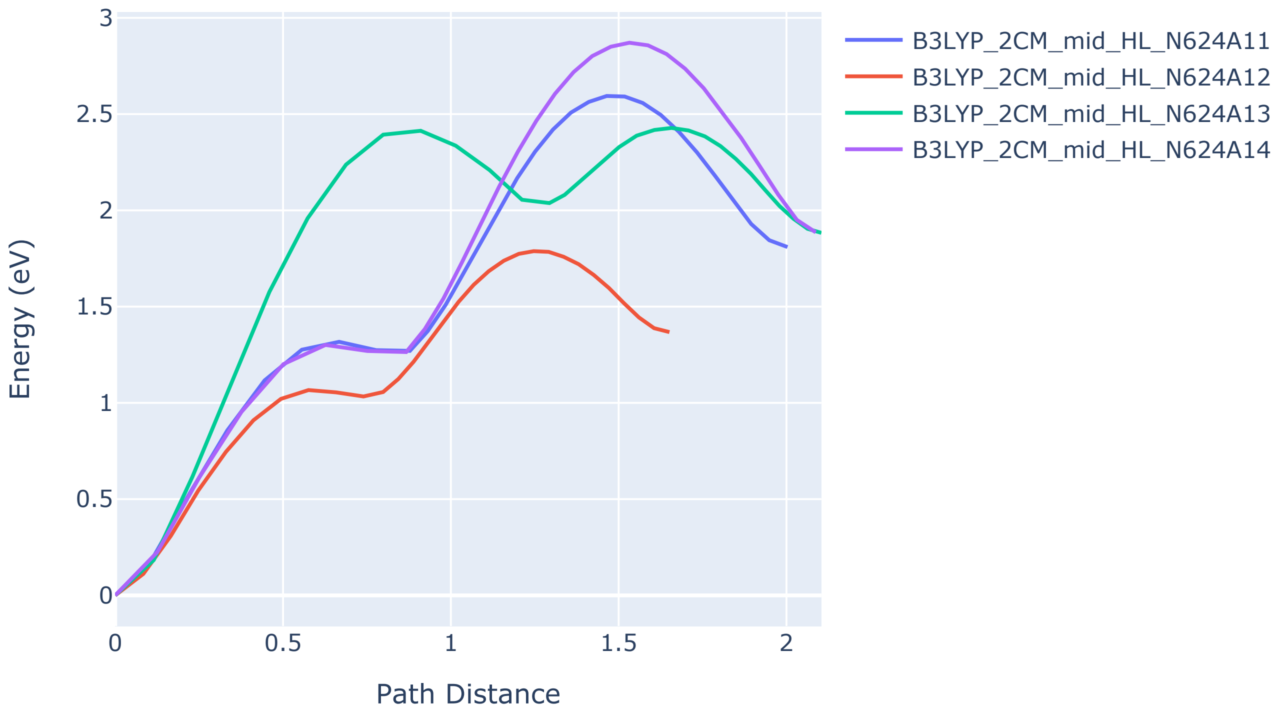

Fig. S14. Minimum energy paths through the instantaneous DPT PES for the PcrA Helicase-DNA complex (base pair N) with the N624A point mutation.

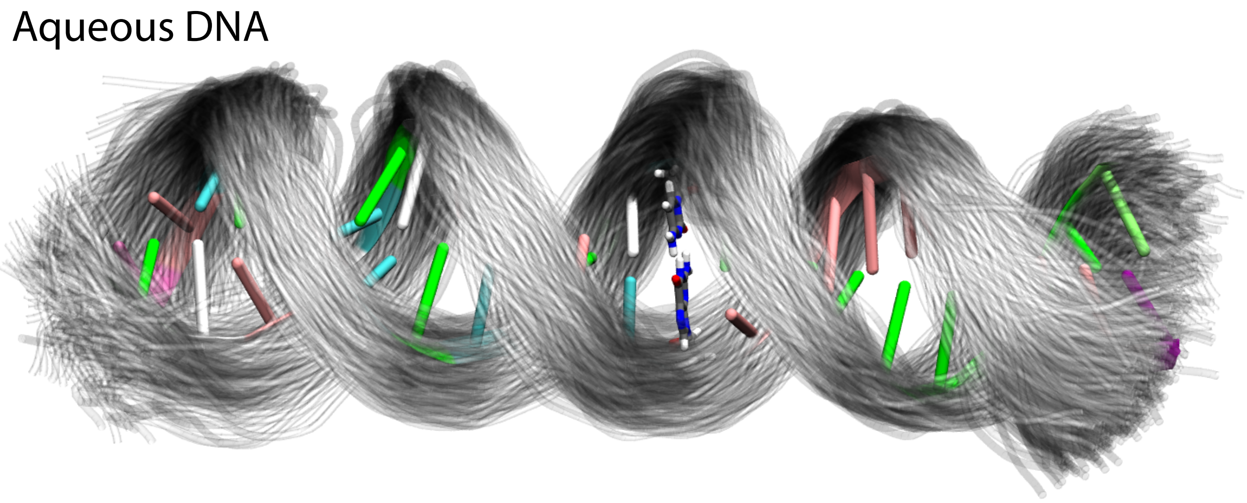

Fig. S15. DNA conformations present during 30ns of NVT MD. One archetypical conformation is shown as a coloured cartoon ladder structure with colours according to the residue: guanine is white, cytosine is cyan, adenine is lime green, and thymine is salmon pink. The GC base pair considered for the DPT is shown as a 'licorice' representation. 600 regularly spaced snapshots from the last 30ns of MD are superimposed as a transparent tube representing the backbone of the DNA.

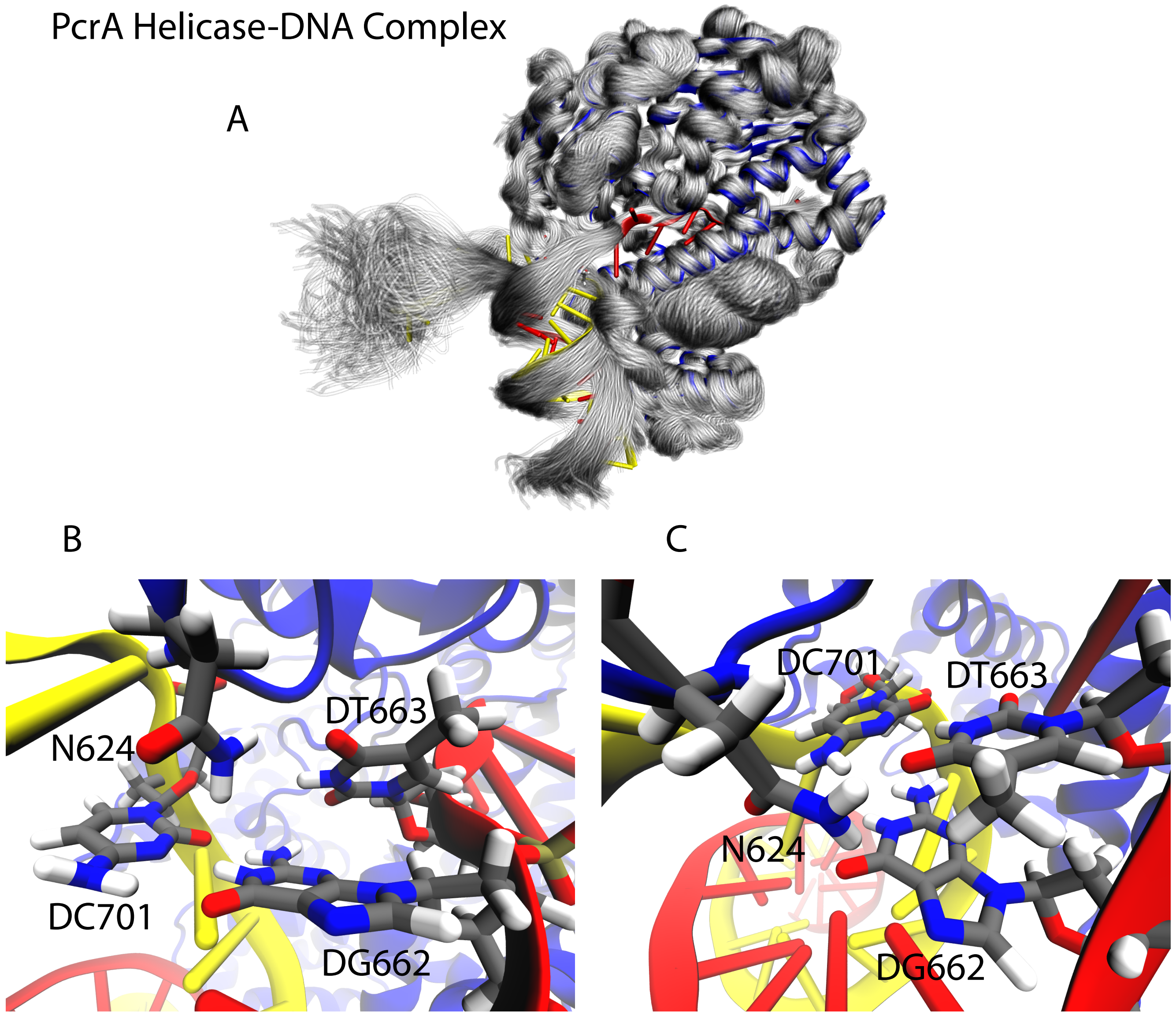

Fig. S16. (A) PcrA Helicase-DNA complex conformations present during 30ns of NVT MD. One archetypical conformation is shown as a coloured cartoon structure with colours according to the chain: DNA is yellow and red, and the protein is blue. 600 regularly spaced snapshots from the last 30ns of MD are superimposed as a transparent tube representing the backbone of the DNA and protein. (B) Primary conformation for the DPT site as used in QM/MM. It can be seen that the asparagine N624's sidechain is in the proximity of the hydrogen bonds between DG662 and DC701. (C) Alternate conformation for the DPT site where the GC tautomerism is presumed irrelevant as DG662 and DC701 have dissociated and a new wobble conformation between DC701 and DT663 is formed. Asparagine N624 has also rotated away from the hydrogen bonds.

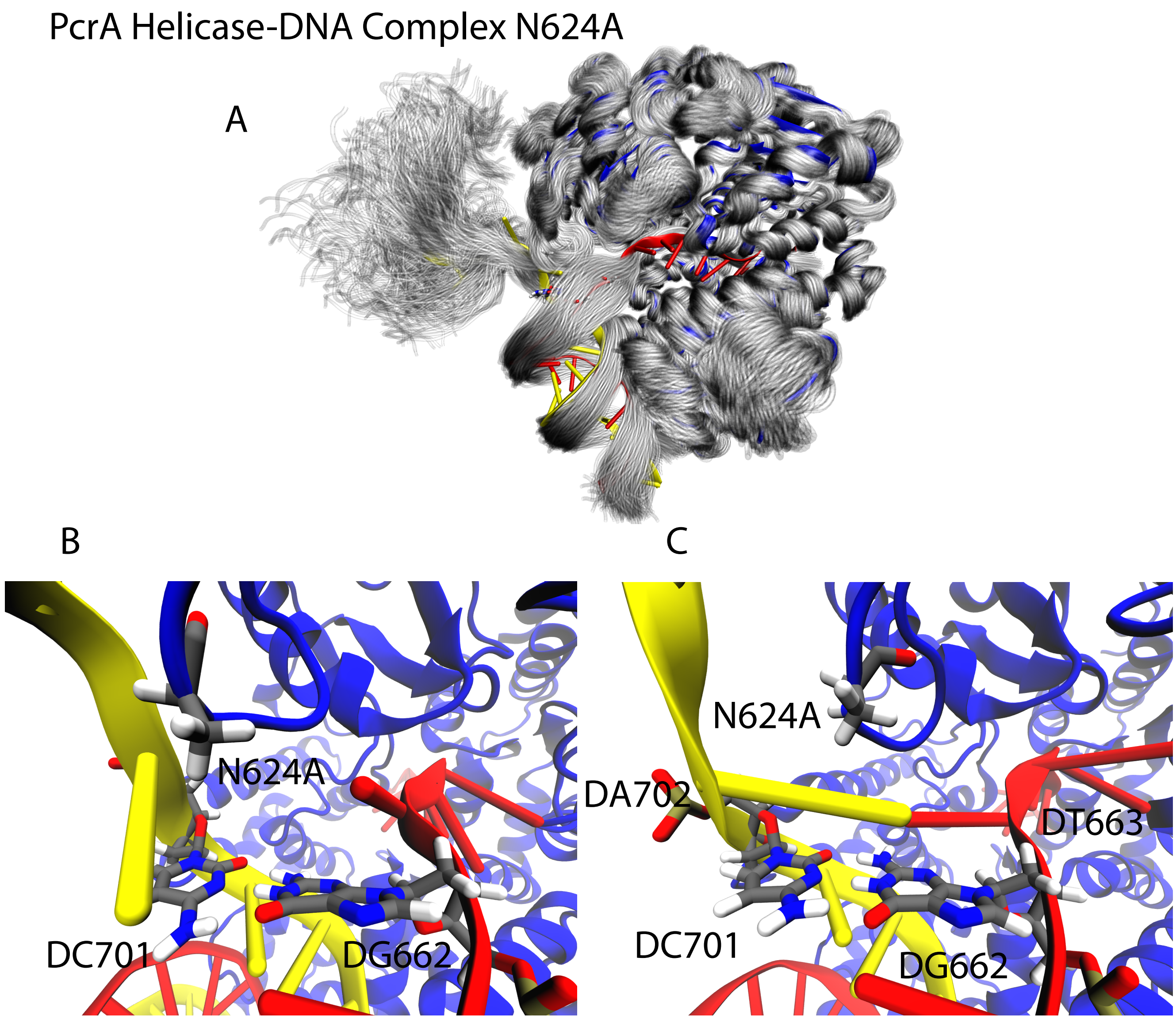

Fig. S17. (A) N624A point mutated PcrA Helicase-DNA complex conformations present during 30ns of NVT MD. One archetypical conformation is shown as a coloured cartoon structure with colours according to the chain: DNA is yellow and red, and the protein is blue. 600 regularly spaced snapshots from the last 30ns of MD are superimposed as a transparent tube representing the backbone of the DNA and protein. (B) Primary conformation for the DPT site as used in QM/MM. The shorter sidechain of the alanine N624A compared to the wild type's asparagine N624 does not interfere with the hydrogen bonding between DG662 and DC701. (C) Alternate conformation for the DPT site where the base pair DG662 and DC701 (base pair N) is no longer the last in the duplex as the pair DT663-DA702 (pair N+1) has reformed.

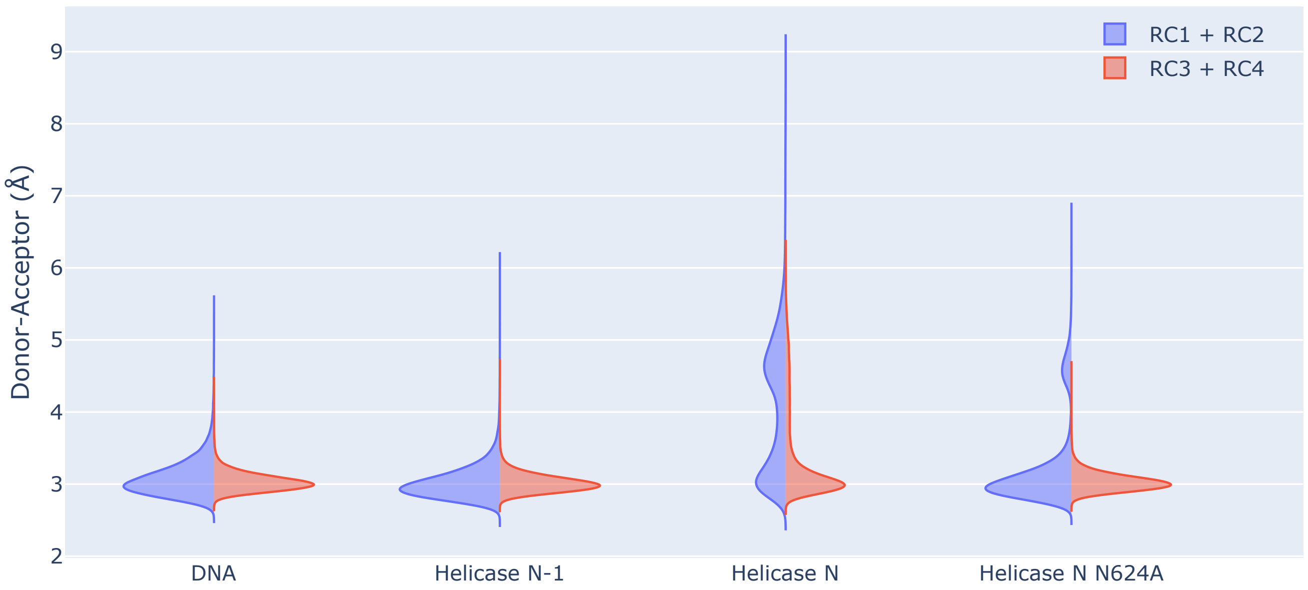

Fig. S18. Donor-acceptor distance statistics for the two hydrogen bonds involved in the DPT of GC in four environments. Each system was simulated for 33ns in NVT Molecular Dynamics.

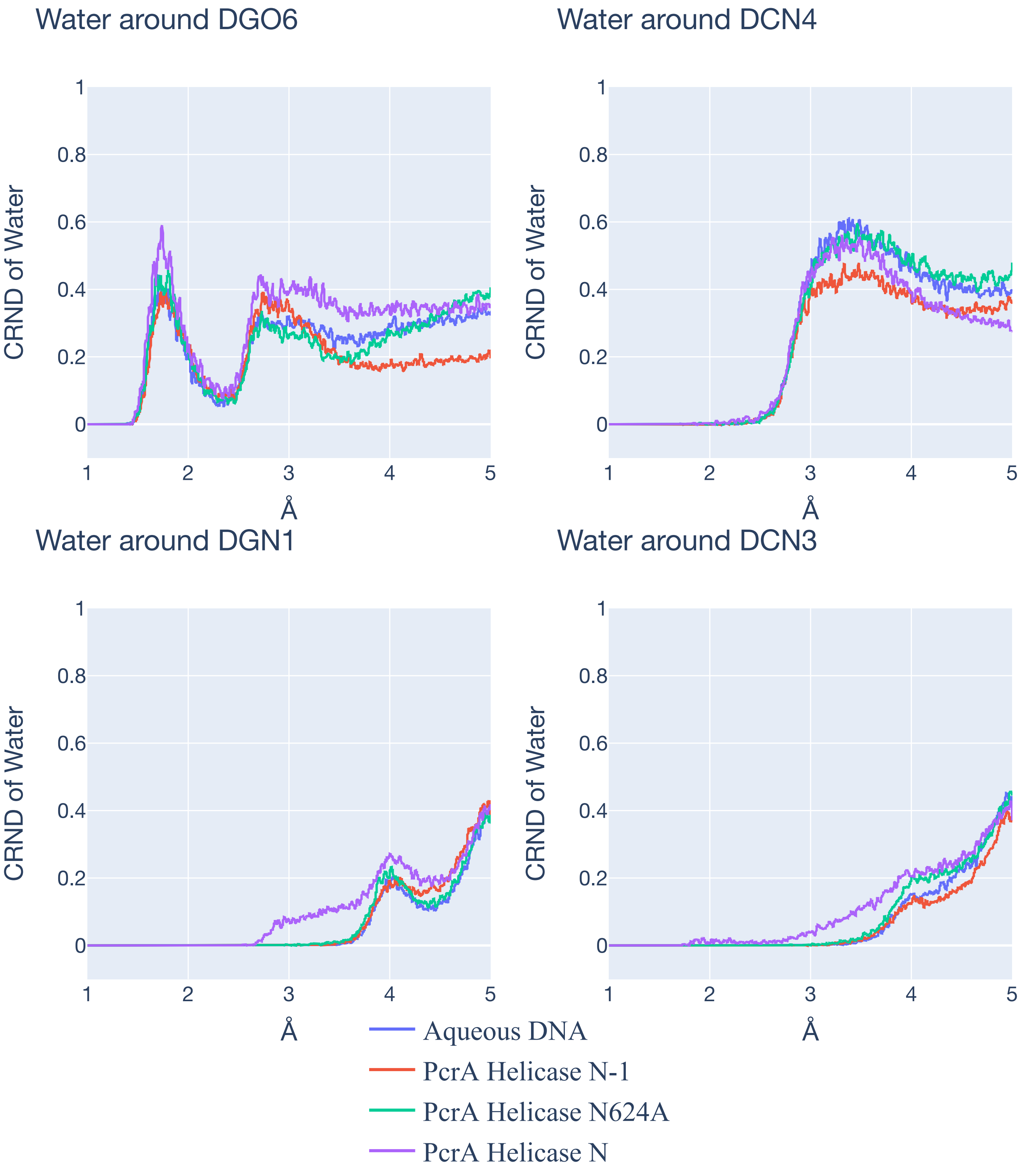

Fig. S19. Radial number density (RND) of water atoms around donor and acceptor atoms involved in the DPT of GC in four environments. Each system was simulated for 33ns in NVT Molecular Dynamics.

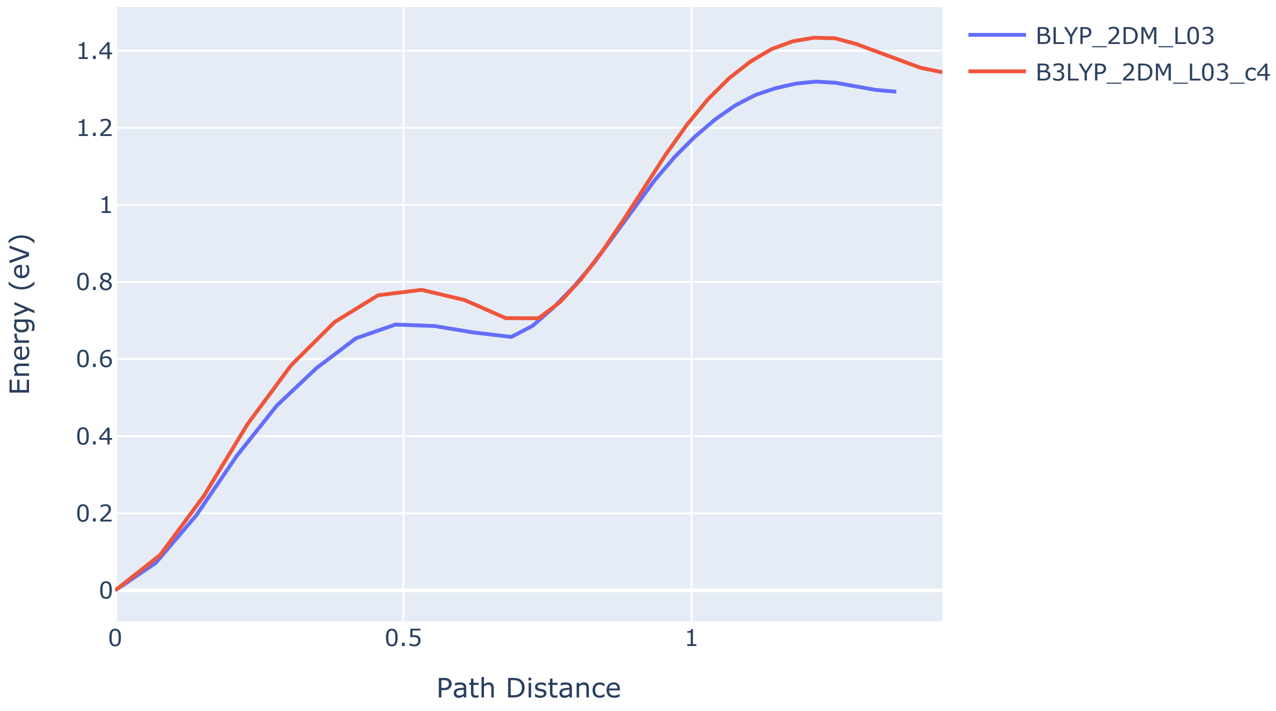

Fig. S20. NEB profiles through instantaneous potential energy surface for the DPT in the same aqueous DNA conformation for BLYP and B3LYP levels of theory.

References

[1] MD Hanwell, et al., Avogadro: an advanced semantic chemical editor, visualization, and analysis platform. *J. cheminformatics* **4**, 1–17 (2012).

[2] RB Best, et al., Optimization of the additive charmm all-atom protein force field targeting improved sampling of the backbone ϕ, ψ and side-chain χ1 and χ2 dihedral angles. *J. chemical theory computation* **8**, 3257–3273 (2012).

[3] J Huang, et al., Charmm36m: an improved force field for folded and intrinsically disordered proteins. *Nat. methods* **14**, 71–73 (2017).

[4] K Hart, et al., Optimization of the charmm additive force field for dna: Improved treatment of the bi/bii conformational equilibrium. *J. chemical theory computation* **8**, 348–362 (2012).

[5] H Berendsen, D van der Spoel, R van Drunen, Gromacs: A message-passing parallel molecular dynamics implementation. *Comput. Phys. Commun.* **91**, 43–56 (1995).

[6] M Winokan, C Vallee, Molparse: A python package for parsing, modifying, and analysis of molecular structure files. (https://github.com/mwinokan/MolParse) (2023).

[7] SS Velankar, P Soultanas, MS Dillingham, HS Subramanya, DB Wigley, Crystal structures of complexes of pcra dna helicase with a dna substrate indicate an inchworm mechanism. *Cell* **97**, 75–84 (1999).

[8] J Yu, T Ha, K Schulten, Structure-based model of the stepping motor of pcra helicase. *Biophys. journal* **91**, 2097–2114 (2006).

[9] J Hutter, M Iannuzzi, F Schiffmann, J VandeVondele, cp2k: atomistic simulations of condensed matter systems. *Wiley Interdiscip. Rev. Comput. Mol. Sci.* **4**, 15–25 (2014).

[10] L Slocombe, M Winokan, J Al-Khalili, M Sacchi, Proton transfer during dna strand separation as a source of mutagenic guanine-cytosine tautomers. *Commun. Chem.* **5**, 144 (2022).

[11] P Virtanen, et al., Scipy 1.0: fundamental algorithms for scientific computing in python. *Nat. methods* **17**, 261–272 (2020).

[12] AH Larsen, et al., The atomic simulation environment—a python library for working with atoms. *J. Physics: Condens. Matter* **29**, 273002 (2017).

[13] L Slocombe, JS Al-Khalili, M Sacchi, Quantum and classical effects in dna point mutations. *Phys. Chem. Chem. Phys.* (2021).
